# Supplementary material for: Direct measurements of interfacial adhesion in 2D materials and van der Waals heterostructures in ambient air
Source: Nat Commun. 2020 Nov 5;11:5607. doi: 10.1038/s41467-020-19411-7 (PMC7645779; doi:10.1038/s41467-020-19411-7)
Supplement: Supplementary file 1 — Supplementary Information [file 41467_2020_19411_MOESM1_ESM.pdf]

Supplementary Information

Direct Measurements of Interfacial Adhesion in 2D Materials and van der Waals Heterostructures in Ambient Air

Hossein Rokni, Wei Lu*

Correspondence to: weilu@umich.edu

This PDF file includes:

Supplementary Note 1	AFM-assisted experimental setup
Supplementary Note 2	Interfacial adhesion energy (IAE) measurements
Supplementary Note 3	Surface roughness measurements
Supplementary Note 4	X-ray photoelectron spectroscopy on pre-annealed MoS_2 crystals
Supplementary Note 5	Comparative studies of IAE
Supplementary Note 6	Interaction of G with airborne contaminants using water contact angle measurements
Supplementary Note 7	Calculations of bending stiffness in 2D crystals
Supplementary Figure 1	Schematic representation of AFM-assisted interfacial adhesion measurements.
Supplementary Figure 2	SEM and AFM images of 2D crystal nanomesas.
Supplementary Figure 3	Attachment of 2D crystal nanomesas to in situ flattened AFM tips.
Supplementary Figure 4	Characterization of microheater devices.
Supplementary Figure 5	Calibration of microheater devices.
Supplementary Figure 6	Steady state thermal simulation of a microheater.
Supplementary Figure 7	Characterization of Peltier-based cooling stage setup.
Supplementary Figure 8	Effect of tilt angle on IAE measurements.
Supplementary Figure 9	Spring constant calibration of AFM probe.
Supplementary Figure 10	X-ray photoelectron spectroscopy of MoS_2 crystal.
Supplementary Figure 11	Rotation and lateral deflection of AFM probe.
Supplementary Table 1	Cohesion energy of carbon nanotubes, few-layer graphene, and graphite.
Supplementary Table 2	Comparisons between calibrated stiffness of probes reported by Koren et al. [15] and the present work.
Supplementary Table 3	Interlayer adhesion energy of carbon nanotubes, few-layer graphene, and graphite on SiO_x.
Supplementary Table 4	Interlayer adhesion energy of MoS_2 on SiO_x.
Supplementary Table 5	Summary of cohesion energy Γ (Jm^{-2}) at intact G, hBN and MoS_2 homointerfaces.
Supplementary Table 6	Interfacial adhesion energy Γ (Jm^{-2}) in similar/dissimilar heterostructures using normal force microscopy.
Supplementary Table 7	Summary of water contact angle measurements and corresponding IAE values of G crystal.
Supplementary References	1-53

Supplementary Note 1. AFM-assisted experimental setup

All AFM measurements were performed under controlled ambient conditions (*T*= 22 °C and 15% relative humidity) by a Park XE-70 microscope which is isolated from mechanical floor vibration by a microscope vibration isolator, and also from acoustic vibration, ambient light disturbance and air flow by a closed box. We determined the noise floor of our AFM setup to be consistently less than 0.3 Å throughout the measurements.

After surface preparation of SiO_x/Si wafer (**Supplementary Note 1.1**), three small pieces of SiO_x/Si substrate were simultaneously loaded onto the AFM stage, including (1) 2D crystal flakes mechanically exfoliated with adhesive tape on microheater arrays which are prefabricated on the SiO_x/Si substrate (piece#1); (2) 25-nm-thick polymer glue (PEDOT:PSS, D-sorbitol) coated on the SiO_x/Si substrate (piece#2), and (3) pre-patterned bulk 2D crystal stamps with 50-100 nm–thick square and circular nanomesas of 55-65 nm in width and diameter, respectively, (**Supplementary Note 1.2**) (piece#3). To minimize the effect of the relative tilt angle, all three pieces were attached to a larger piece of SiO_x/Si substrate pre-coated by the ultrathin glue film (PEDOT:PSS, D-sorbitol), followed by placing the larger piece onto a multistage Peltier cooling element equipped with a tilt control mechanism (angle resolution: ±0.5°) beneath the cooling stage. Instead of immediately removing all 2D crystal-loaded adhesive tapes from the piece#1 to complete the mechanical exfoliation onto the microheaters, we only peeled off the tape containing the 2D crystal flakes of interest for the interfacial adhesion measurements, thereby enabling much better control over the possible adsorption of airborne contaminants onto the fresh surface of 2D crystals.

For the AFM force-displacement measurements, a highly doped silicon AFM probe (NANOSENSORS^{TM}, ATEC-FM, with a nominal spring constant of 2.8 Nm^{-1} and a typical tip radius of curvature better than 10 nm) was used where the tip is positioned at the very end of the cantilever and pointing outward which provides a more accurate positioning of the tip apex. Since our experiments require a flat plateau at the apex parallel the piece#1 surface on which all interfacial adhesion measurements were conducted, we scanned the tip in contact mode on its SiO_x surface to achieve an atomically flat surface with an RMS roughness of <1 nm. The *in situ* flattened tip was next moved from piece#1 to piece#2 and coated with a very thin layer of polymer glue by putting the tip apex in gentle contact with the PEDOT:PSS(D-sorbitol) film. For the precise attachment of 2D crystal nanomesa to the glue-coated flattened apex, the tip was moved from piece#2 to piece#3, followed by locating the nanomesas by the non-contact AFM topography measurements. The glue-coated tip apex was then moved to the center of the selected 2D crystal nanomesa at an applied load of 2 nN and held in contact with the nanomesa for 10 mins. Afterwards, the tip was gently pulled away from the substrate surface in a direction perpendicular (parallel) to the single basal plane of 2D crystal, leading to pulling off (shearing) the upper section of the nanomesa (attached to the tip apex) from the lower section (fixed to the 2D crystal substrate) (**Supplementary Note 1.3**).

To study the effect of thermal annealing on the interfacial adhesion, the flakes were heated up to 300 °C by applying a constant current to the microheater (**Supplementary Note 1.4**). Using the microheater can not only significantly alleviate the adverse effect of high temperature on the AFM probe by locally heating the substrate but also provide a uniform temperature distribution over the heated 2D flakes which are in direct contact with the heating lines. For each temperature change, enough time was given to the flakes to reach steady-state temperature (*T*). Then, the 2D crystal tip was engaged with the sample surface at a pressure of 5 MPa (unless otherwise noted) for 15 min to reach thermal equilibrium and then the substrate cooled back down to room temperature to perform the interfacial adhesion measurements. Similarly, we conducted a series of the interfacial adhesion measurements at subzero temperature by first cooling the substrate surface down to -15 °C using a multistage Peltier element and then removing the 2D crystal-loaded adhesive tape from the piece#1 (**Supplementary Note 1.5**).

During the attachment of nanomesas to the glue-coated tip, *F–d* curves can be recorded as the tip is gently pulled away from the substrate surface in a direction perpendicular to the single basal plane of 2D crystal, leading to pulling off the upper section of the nanomesa (attached to the tip apex) from the lower section (fixed to the 2D crystal substrate) (**Supplementary Fig. 1a**). Similarly, the shear force-displacement curves are recorded as the nanomesa is sheared along the long axis of the cantilever tip rather than perpendicular to its long axis (as is the case in the conventional lateral force microscopy) to obtain more accurate shear force measurements (**Supplementary Fig. 1b**). Also, the interfacial adhesion measurements at untreated and treated interfaces were performed upon

retraction of the 2D crystal tip from the untreated and precooling treated surfaces (**Supplementary Fig. 1c** and **1d**, respectively).

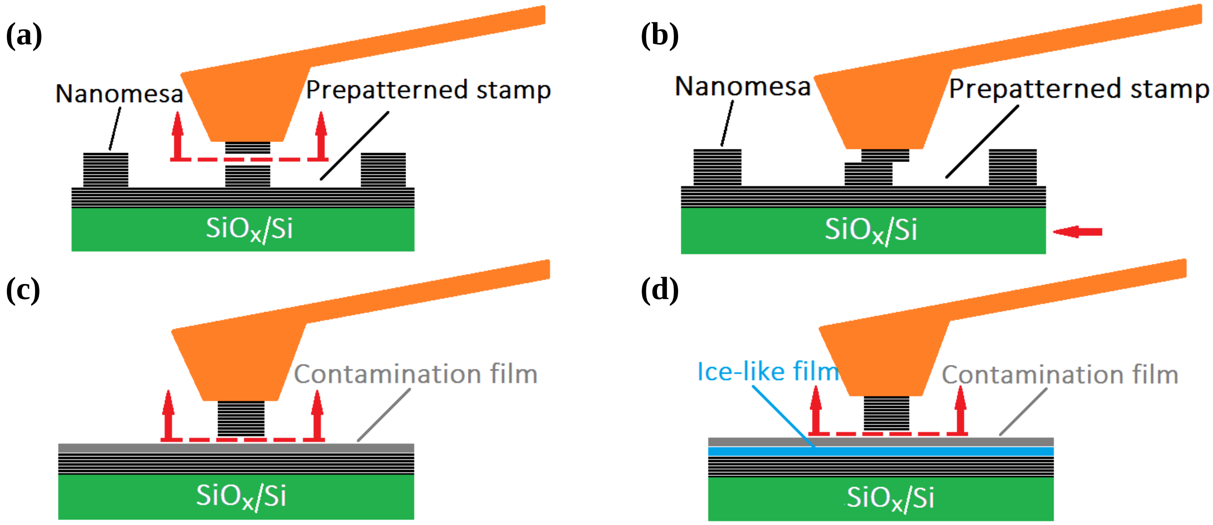

Supplementary Figure 1. Schematic representation of AFM-assisted interfacial adhesion measurements. (a) Normal and (b) shear interfacial adhesion measurements at the intact homointerfaces. (c) Interfacial adhesion measurements at the (c) untreated and (d) precooling-treated homointerfaces.

Supplementary Note 1.1. Sample preparation

Surface preparation of SiO_x substrates: Prior to mechanical exfoliation of 2D crystal flakes, square pieces of a 300-nm-thick SiO_x/Si wafer were ultrasonically cleaned in sequential baths of acetone, isopropanol and deionized water and dry blown with nitrogen.

2D crystal preparation: G, hBN and MoS_2 flakes were prepared by mechanical exfoliation of ultrapure single crystal of hBN, highly oriented pyrolytic graphite (HOPG) and single crystal of MoS_2 on the SiO_x/Si substrate.

Supplementary Note 1.2. Fabrication of nano-sized 2D crystal mesas

A ~100-nm-thick bilayer of polymethyl methacrylate (PMMA) 495K (60 nm)/950K (40 nm) is spin coated onto the freshly cleaved surface of 1-mm-thick HOPG (SPI, Grade 1, with a mosaic spread value of 0.4^o), hBN (grade A, with single crystal domains over 100 μm) and MoS_2 (429MS-AB, natural single crystals from Canada) substrates, baked each layer for 10 min at 120 °C to evaporate the solvent and then patterned by electron beam lithography. The mask was designed in such a way that arrays of G, hBN and MoS2 nanomesas were fabricated with both square and circular cross sections and both shapes were used in interfacial adhesion measurements. After developing the exposed PMMA area in 1:3 MIBK/NMP, a 10-nm-thick aluminum film is deposited by thermal evaporation, followed by lift-off process in acetone. The unprotected HOPG, hBN and MoS_2 areas are thinned down by using a reactive ion etching system with pure O_2 (precursor flow rate: 10 sccm, RF power: 40W, pressure: 10 mTorr), $CHF_3/Ar/O_2$ (10/5/2 sccm, 30W, 10 mTorr) and SF_6 (20 sccm, 100W, 20 mTorr) reactive gases, respectively. Square (circular) mesas with a width (diameter) of 55-65 nm and etch depth of 50-100 nm emerge from 2D crystal substrates during the plasma etch.

After plasma etching, the sample is soaked in 0.1 mol/l KOH water solution for ~3 min to remove the Al layer, followed by an annealing process at 200-600 °C under constant Ar/H_2 flow for one hour to remove any resist/metallic residues from 2D crystal substrates. **Supplementary Fig. 2a** shows the SEM images of MoS_2 square and G circular nanomesa arrays. The corresponding 3D AFM image of MoS_2 square nanomesas is also shown in **Supplementary Fig. 2b**. The sidewall profile in the AFM images is steep (as also confirmed by the height profiles

in **Supplementary Fig. 2c**), indicating that a low etch rate of 15 nm/min for the fabrication of the mesa structures results in a minor sidewall taper toward the substrate.

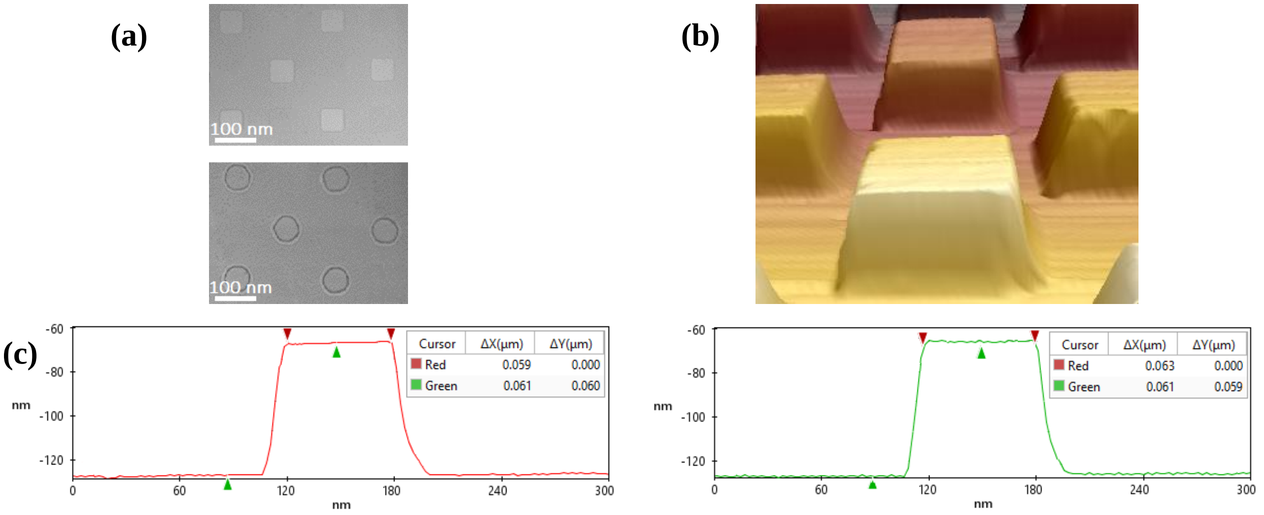

Supplementary Figure 2. SEM and AFM images of 2D crystal nanomesas. (a) SEM images of MoS_2 square and G circular nanomesa arrays. (b) 3D AFM image of MoS_2 square nanomesas. (c) Corresponding height profiles of 60-nm-deep MoS_2 square mesas with an average width of 60 nm.

Supplementary Note 1.3. Attachment of 2D crystal nanomesas to *in situ* flattened AFM tip

Since the attachment of 2D crystal mesas to the tip apex requires a well-defined and smooth landing area, the method described in [1] was adopted to flatten the AFM tip by scanning it in contact mode on the SiO_x/Si substrate. The morphology and area of the flat tip were first quantified through SEM and AFM images (**Supplementary Fig. 3a, b**). Next, the

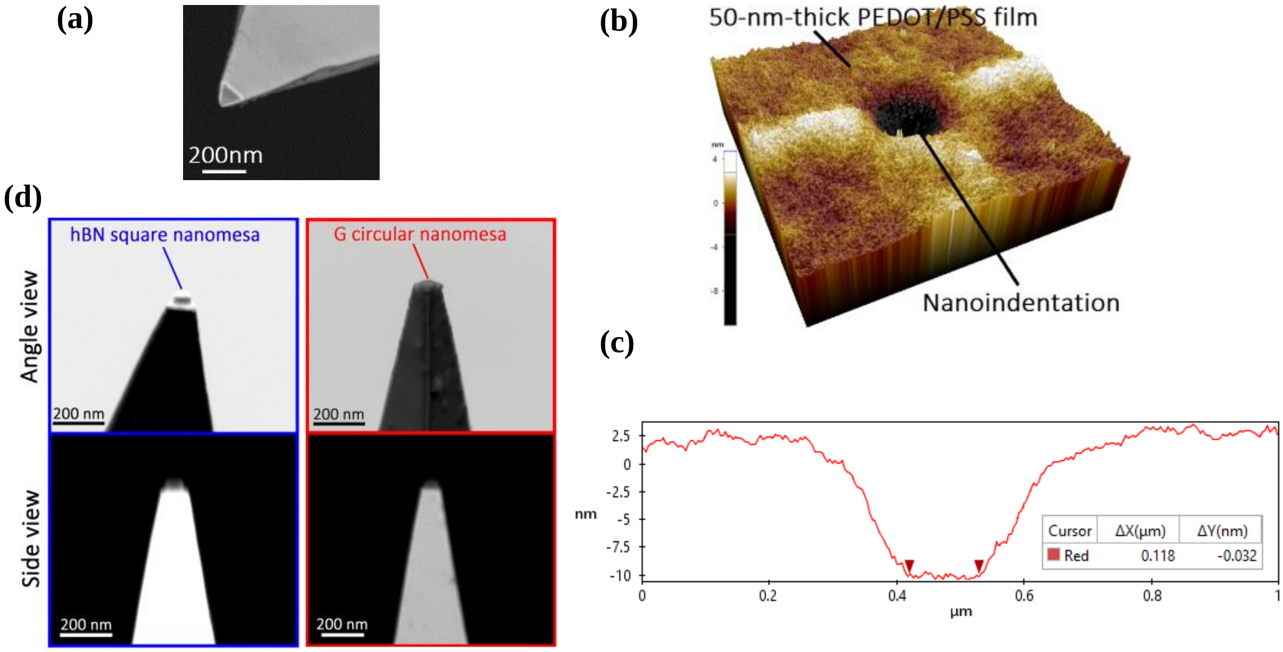

Supplementary Figure 3. Attachment of 2D crystal nanomesas to *in situ* flattened AFM tips. (a) SEM image of an in situ flattened tip apex. (b) AFM topography image of nanoindentation of the tip apex into the 50-nm-thick PEDOT/PSS film on the SiO_x/Si substrate. (c) Indented profile of the tip shows a very flat and smooth surface. (d) Typical SEM images of the tip with an attached square or circular nanomesa.

surface roughness and the contact area of the tip apex were determined by imprinting the tip onto the 50-nm-thick PEDOT/PSS film, followed by the AFM tapping-mode imaging. The AFM indent profile of the tip confirms a very flat triangular shape with an RMS roughness of less than 0.5 nm (**Supplementary Fig. 3c**). After glue coating the flattened apex by making a contact between the tip and piece#2, we moved the tip from piece#2 to piece#3 while switching the operational mode of the AFM to non-contact mode to determine the location of each nanomesa. Although the tip apex is flat, the non-contact mode can still provide us with desired resolution imaging for the subsequent attachment of the nanomesa to the tip apex. Switching the mode of operation back to the contact mode, the glue-coated tip apex was then moved to the center of the selected 2D crystal nanomesa and held in contact with the nanomesa at an applied load of 2 nN for 10 mins. Afterwards, the tip was gently pulled away from the substrate surface in a direction perpendicular (parallel) to the single basal plane of 2D crystal, leading to pulling off (shearing) the upper section of the nanomesa (attached to the tip apex) from the lower section (fixed to the 2D crystal substrate). **Supplementary Fig. 3d** shows typical SEM images of tip-attached square and circular nanomesas. In order to replace an old nanomesa with a new one, the attached nanomesa is simply removed by gently rubbing the AFM tip on the SiO_x surface under an applied contact load of 200 nN, followed by re-coating the flattened tip using an ultrathin glue film (25 nm in thickness) and attaching the new nanomesa using the same technique as described above.

Supplementary Note 1.4. Fabrication and characterization of microheaters

In order to perform AFM measurements at elevated temperatures, an external stage heater (e.g., silicone rubber heater, Kapton heater, etc.) is widely used to heat a sample. However, microdroplets of condensed water or volatile components in the atmosphere and/or emitted from the hot sample may condense on the relatively cooler cantilever surface (being typically 10-20 μm away from the heated sample makes the probe cooler than the sample surface), as shown in **Supplementary Fig. 4a**. Such microdroplets can induce adverse effect on the deflection and oscillation amplitude measurements of the probe by scattering the laser beam and also on the physical properties of the probe by changing its resonance frequency and spring constant. As a remedy, we used microheaters to confine the heat to a micron-sized region on the SiO_x/Si substrate via the Joule effect with very low power consumption.

Microheater array was fabricated by the deposition of Ti(20 nm)/Pt(200 nm) stack on the 300-nm-thick SiO_x insulator by RF sputtering, followed by a lift-off step to pattern heating lines and contact pads. While large heating areas can be achieved by meander or spiral shaped microheaters, heating lines will be partially or fully covered by the subsequent exfoliated flakes, rendering the microheaters useless. As such, a simple U-shaped geometry was used to yield more 2D crystal flakes in direct contact with the heating lines, enabling a uniform heat distribution over the entire area of the flakes with almost the same temperature as the heating lines. **Supplementary Fig. 4b** shows the SEM image of the fabricated microheater device with surrounding 2D crystal flakes, indicating very smooth and uniform line width (5 μm) of the heating element with 15 μm spacing between the lines. Fine gold wires were used for connecting the contact pads to a programmable dc power supplier (Instek PSP-405) through package pins.

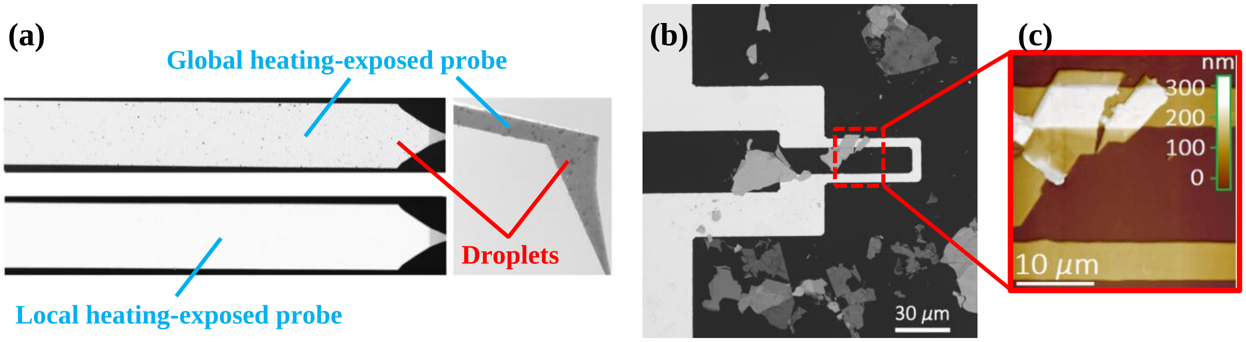

Supplementary Figure 4. Characterization of microheater devices. (a) SEM images of the probe exposed to a temperature of 95 °C using a 1cm×1cm Kapton heater (i.e., global heating) and a micron-sized heater (i.e., local heating). (b) SEM image of a representative microheater surrounded by the exfoliated MoS_2 flakes. (c) AFM

noncontact-mode image of the region marked by the square in (b), taken by the G crystal tip.

For calibration purpose, a Probe station, consisting of a microscope, a micromanipulator with a set of ultrasharp needle probes, resistance measurement unit along with an integrated hot chuck (having an accuracy of 1 °C) was used to measure the electrical resistance change of the microheaters as a function of temperature ranging from RT to 300 °C. The resistance measurements were repeated for ten microheaters, all showing a self-consistent linear dependence of the electrical resistance change on temperature with the best linear fit of R=0.058T+21.15 Ω, which is consistent with the following resistance-temperature expression $R=R_0+R_0\alpha_0(T-T_0)$, where R_0 and α_0 are the resistance of the microheater and the temperature coefficient of resistance of Pt, respectively, at room temperature T_0. The microheaters were subsequently characterized by Joule heating with a DC power supply of 1 to 10 mW input power, followed by measuring the electrical resistance of the microheaters using a digital multimeter. We again observed a linear dependence of the resistance on the input power P with the best linear fit of R=1.613P+22.50 Ω. Assuming that the electrical resistance of the microheaters obtained from the hot chuck measurements is equal to that obtained from the Joule heating measurements, the linear relationship between the input power and the temperature is given by T=27.81P+23.28 °C (**Supplementary Fig. 5a**). This equation was further verified by temperature measurements in close proximity to the microheaters by an infrared camera during Joule heating, as shown in **Supplementary Fig. 5a**.

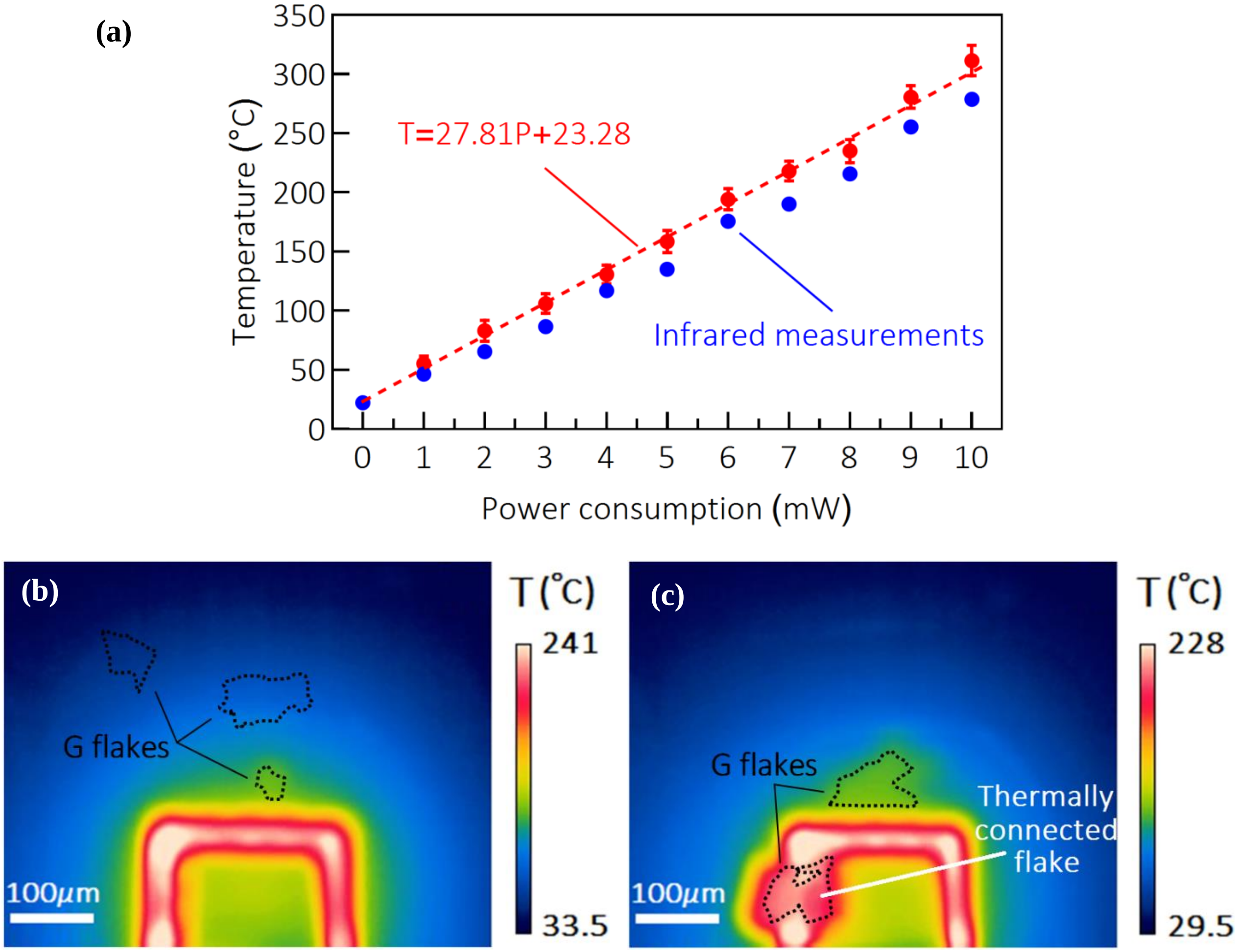

Supplementary Figure 5. Calibration of microheater devices. (a) Calibration of the microheater devices using the hot chuck/Joule heating (red dots) and IR camera (blue dots), both showing a linear relationship between the temperature and the input power. The dashed red line is linearly fitted to the combined hot chuck/Joule heating results. (b, c) IR results for the temperature distribution of the microheaters with the thermally-disconnected (b) and connected (c) G flakes.

Slightly lower temperatures measured by the IR camera may be attributed to the aperture-limited resolution of the camera that does not allow identifying the hottest spot in the microheater devices. Our transient measurements obtained by the application of a constant voltage pulse to the microheaters for a very short time also revealed a very fast thermal response (i.e., 100 ms from RT to 300 °C). It should be noted that the microheaters are mostly fabricated on a thin membrane to reduce the power consumption; however, our microheaters on the silicon substrate avoid deformation of the membrane during the approach/retraction process of the AFM tip, making adhesion measurements more accurate at a cost of higher power consumption.

After the calibration of the microheaters, we note that the temperature of 2D crystal flakes strongly depends on their distance from the heating source. This can be understood by examining the radially symmetric temperature decay of a thin silicon membrane with the heating laser spot at its center which is a logarithm function of the distance as follows

$$T(r) = T_0 - \frac{P}{2\pi dk} \ln \frac{r}{r_0} \tag{1}$$

where P is the input power, d is the thickness of the membrane, k is the thermal conductivity of Si and (T_0, r_0) is a reference point in the temperature field. Hence, we used IR camera to map the temperature distribution within the mechanically exfoliated 2D crystal flakes. For clarity in the IR images, we scaled up the microheaters from 25×55 μm^2 to 250×550 μm^2 heating area while preserving all the geometric features. We observed that the flakes exfoliated few microns away from the heating lines possess much lower temperature than the microheaters and may not be well detected under the IR camera (**Supplementary Fig. 5b**) while those in direct contact with the heating lines display almost the same temperature (**Supplementary Fig. 5c**), thanks to very high thermal conductivity of 2D crystals. As a result, only flakes exfoliated onto the heating lines were considered for the interfacial adhesion measurements at elevated temperatures.

To further evaluate the temperature of the microhetaer, 2D crystal flakes and the probe, steady state temperature distribution of our AFM setup needs to be calculated. As such, a 3D model of the setup was created using COMSOL Multiphysics (**Supplementary Fig. 6a**) to numerically solve the following thermal transfer equation

$$\nabla.(-k\nabla T) + \rho c \frac{\partial T}{\partial t} = q_s \tag{2}$$

where k, ρ and c are the thermal conductivity, material density and specific heat capacity, respectively; and T and q_s are the temperature and the density of the heat power generation, respectively. The microheater, graphite flakes, SiO_x/Si substrate and the AFM probe were surrounded by an air block of 1400×1400×420 μm^3. In the desired temperature range (<300 °C), the resulting radiation losses are negligible due to the very low emissivity of the materials involved and thus heat losses caused by the thermal convection and conduction were taken into account. Moreover, all sides of the air block were assigned a symmetry boundary condition. **Supplementary Fig. 6b** shows the simulated temperature distribution of the G flakes and the microheater at an input power of 5 mW. Consistent with our experimental observations, while the temperature of G flakes surrounding the microheater is highly smaller than that of the heating lines, the flake in direct contact with the microheater shows the same uniform temperature distribution as the heating line does.

We also probed temperature distribution of the AFM tip in contact with the flake at 221 °C. **Supplementary Fig. 6c** demonstrates that the localized heating of 2D crystal flakes can effectively restrict the heat flow of the AFM tip only through the 2D crystal nanopillar with the cross-sectional area of ~0.003 μm^2, thereby maintaining the cantilever shank at temperatures consistently less than 70° C (**Supplementary Fig. 6d**), thanks also to the protruded (3.2 μm from the very end of the cantilever) and tall (18.5 μm) tip which also maximizes the separation between the cantilever shank and the microheater.

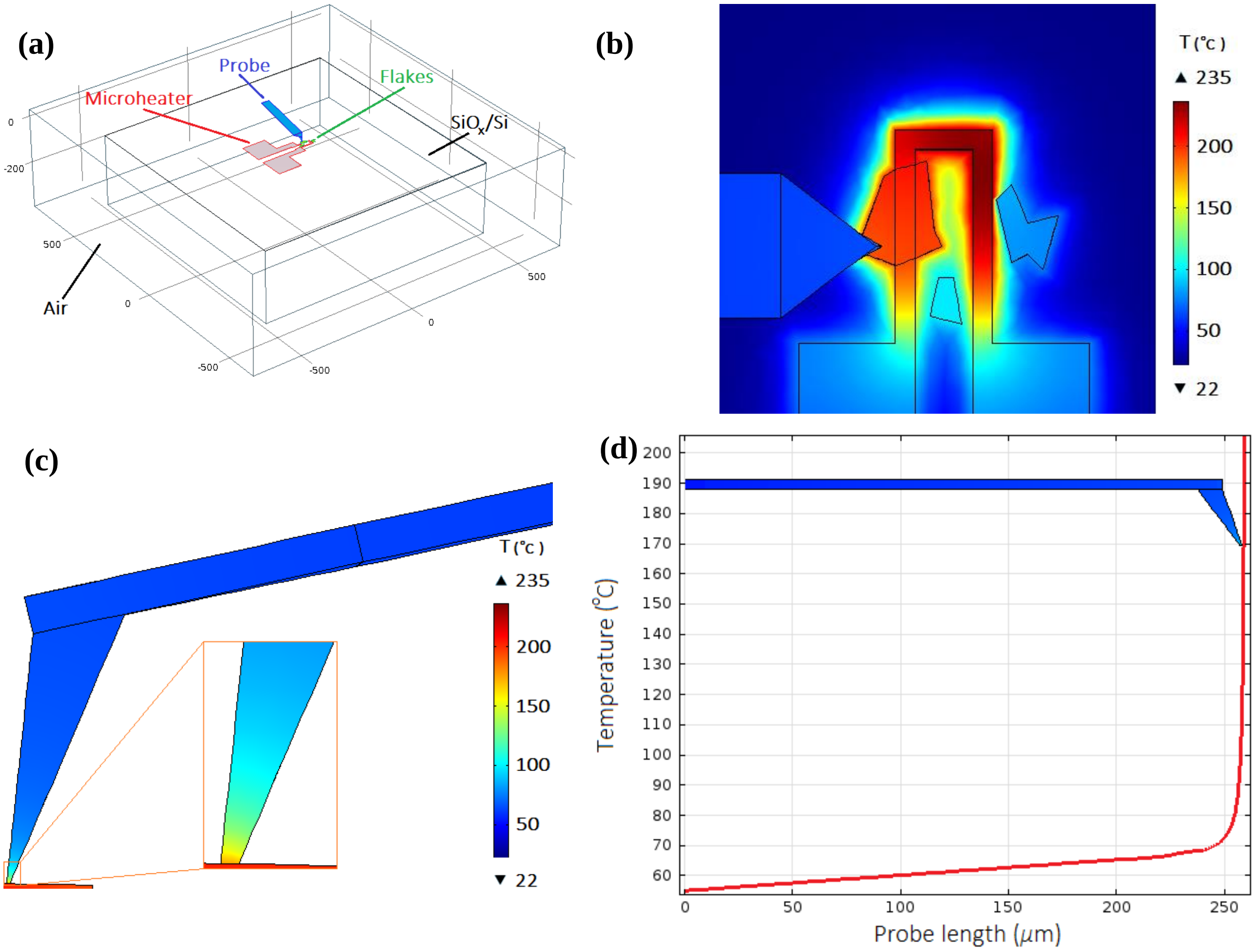

Supplementary Figure 6. Steady state thermal simulation of a microheater. (a) 3D model of the AFM setup developed in COMSOL. (b) Steady state thermal simulation of the G flakes and microheater over the SiO_x/Si substrate at an input power of 5 mW. (c) Temperature distribution of the AFM probe. (d) Temperature distribution profile along the probe length.

Supplementary Note 1.5. Cooling stage setup

The home-built cold stage is based on a multistage Peltier element which is attached to a machined aluminum spacer and connected to a dc power supply, where the spacer with a magnet in the Teflon base is securely fixed to the magnetic sample holder of a variable tilt mount on the X-Y piezo stage (**Supplementary Fig. 7a**). The Teflon base is used to further protect the piezo stage against overheating. A piece of PEDOT:PSS(D-sorbitol)-coated SiO_x/Si wafer is then attached to the cooling element using metal spring clips, where thermal compound spreads at their interface to improve the heat transfer. The aluminum spacer alone enabled to further extract the heat from the hot side of the Peltier element, thereby cooling the surface of small SiO_x/Si pieces, which are glued with PEDOT:PSS(D-sorbitol) to the SiO_x/Si substrate, down to -15.2 °C (**Supplementary Fig. 7b**).

Upon turning on the Peltier cooling element, microdroplets of water in the atmosphere will immediately condense on the substrate surface, increase in size and merge together to form bigger droplets and eventually cover the entire substrate surface. After turning off the Peltier, the microdroplets start to evaporate immediately at room temperature. **Supplementary Figs. 7c-e** show the condensation and evaporation process of microdroplets under the optical microscope within a short time period of 10 sec.

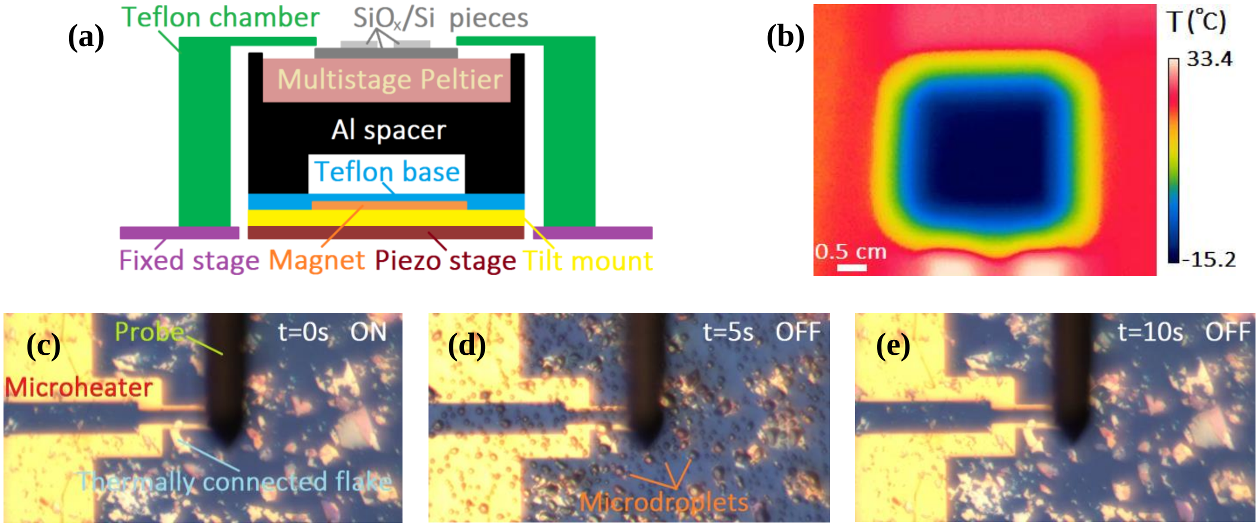

Supplementary Figure 7. Characterization of Peltier-based cooling stage setup. (a) Schematic of Peltier-based cooling stage setup. (b) Infrared image for the temperature distribution of the SiO_x/Si pieces on the multistage Peltier element, showing a very uniform temperature of -15.2 °C. (c)-(e)Wetting behavior during condensation and evaporation of water microdroplets on the surface of the mechanically exfoliated G/SiO_x/Si substrate when the Peltier cooling element is (c) turned ON at 0 sec, (d) then turned back OFF right after 5 sec and (e) kept in the OFF state for another 5 sec.

Supplementary Note 2. Interfacial adhesion energy (IAE) measurements

All retraction *F–d* curves between 2D crystal tips and untreated/precooling-treated substrates were obtained under controlled ambient conditions in the near-equilibrium regime. For each 2D crystal substrate, we considered 15 thermally-connected crystal flakes on each of which 10 individual adhesion measurements at a contact pressure of 5 MPa (unless otherwise noted) were taken from different locations of the flake surface at each annealing temperature to confirm the reproducibility. The contact time (dwell time) of 2D crystal tips with the substrate was 15 min to reach thermal equilibrium at the contact interface. The approach speed was set to be 10 nm/s while a very slow pulling rate of 1 nm/s was used so that the tip remains in thermodynamic equilibrium with the substrate upon tip retraction. Such a slow pulling rate was achieved by using a 16-bit digital-to-analog converter in low voltage mode with an ultralow noise AFM controller which significantly improved the Z scanner's vertical resolution to 0.1 Å at the expense of limiting the Z scanner's motion range. Very careful adjustment of the Z servo gain to suppress any possible oscillation of the Z scanner combined with an ultralow noise floor (<0.3 Å) in our setup could further make the retraction measurements in the near-equilibrium regime possible. In order to measure the cohesion energy, during the attachment of nanomesas to the glue-coated tip, *F–d* curves were recorded as the tip was gently pulled away from the substrate surface in a direction perpendicular (parallel) to the single basal plane of 2D crystal, leading to pulling off (shearing) the upper section of the nanomesa (attached to the tip apex) from the lower section (fixed to the 2D crystal substrate). The annealing temperature for the case of cohesion measurements (studied after completion of our interfacial adhesion experiments) was controlled by a Kapton heater while the probe was fully retracted (~ 4 cm).

In order to identify whether the *F–d* curves are measured at the interface of the tip-attached 2D crystal nanomesa and the sample or within the thickness of the tip-attached 2D crystal nanomesa, we first measured the intrinsic cohesion energy of 2D crystals (**Fig. 1e** and gray circles in **Fig. 2a**), confirming that the cohesion energy across the 2D crystal nanomesa is larger than the interfacial adhesion energy at all 2D crystal tip-sample interfaces. We also observed larger pull-off forces at the intact interfaces compared to contaminated interfaces, well consistent with our reported IAE values. Therefore, the separation most likely takes place at the tip-sample interface rather than somewhere across the thickness of the tip-attached 2D crystal nanomesa. Moreover, for each tip-attached nanomesa, we formed all contacts with 1 μm interval spacing within the same distance from the heating line, allowing us to

easily locate and scan all contact spots (using the non-contact AFM mode) for any possible exfoliation of monolayer or few layers of 2D crystal from tip-attached 2D crystal nanomesa onto the sample. For the contact spot with exfoliated mono/few-layer 2D crystal, the area under the corresponding *F–d* curve was considered as the intrinsic cohesion energy rather than the interfacial adhesion energy at the tip-sample interface.

Supplementary Note 2.1. Calculation of IAE and cleavage/shear strength from *F-d* curves

While the adhesion forces were calculated by the calibrated spring constant and the measured deflection signal of the AFM probe, the IAE per unit area (Γ, Jm^{-2}) was calculated by integrating the retraction force as a function of the piezo displacement, followed by dividing the resulting adhesion energy by the known contact area at the interface. The reason behind using the area under the force-piezo displacement curve is that for stiff interfacial contacts, the external energy required to overcome the internal adhesion energy consumed/dissipated at the interface is presumed to be equal to the bending energy stored in the cantilever. In fact, we did not measure the deformation of the sample as 2D nanomesas and substrates are almost rigid. Rather, we measured the separation energy as the nanocracks start to form due to the localized nano delamination during the retraction process and propagate at the separation plane until the complete separation takes place [1]. As we already discussed in detail in the main text, the crack propagation at the contact interface results in the separation not the deformation does. This can also be immediately confirmed by our MD simulations in the main text where a tiny distance (~0.3 nm) between two adjacent layers results in full separation at the interface with negligible deformation. It is also worth pointing out that the reported distance between the initiation of the separation and full separation of the tip-sample in **Fig. 1b** is the piezo displacement (e.g., ~10 nm at hBN/hBN, ~9 nm at G/G and ~5 nm at MoS_2/ MoS_2 interfaces) rather than the interlayer distance between two adjacent 2D crystal layers and thus does not represent the distance of short-range vdW interaction at the tip-sample interface.

In order to extract the interfacial adhesion energy from the shear force-displacement curves, we first assumed a lateral shear force F_s being applied to a square mesa of width w or a circular mesa of diameter *D*, leading to the lateral displacement *x* of the upper section relative to the bottom section of the mesa and creation of new interface area $A(x)$. At the sliding interface, the total free energy may change by $U(x) = -\Gamma A(x)$. We next obtained the corresponding interfacial adhesion force opposing new surface formation as $F_a(x) = -\,dU(x)/dx = \Gamma\, dA(x)/dx$. For the circular/square mesa with the following new interface area

Circular mesa: $$A(x) = \frac{D^2}{2}\left[\cos^{-1}\left(\frac{x}{D}\right) - \frac{x}{D}\sqrt{1-\left(\frac{x}{D}\right)^2}\right]$$

Square mesa: $$A(x) = w(w-x)$$

the corresponding interfacial adhesion forces can be written as

Circular mesa: $$F_a(x) = -\Gamma D\sqrt{1-\left(\frac{x}{D}\right)^2}$$

Square mesa: $$F_a = -\Gamma w$$

By inspection, one can see that the maximum interfacial adhesion force, F_s^m, required to initiate sliding the circular and square mesas can simply be given by ΓD and Γw, respectively.

While the cleavage strength can be obtained by $\sigma_{33} = P/A$, where P is the pull-off force and A is the interface area, by definition of the interlayer shear strength at the sliding interface, $\tau_s = F_s^m/A$. As a result, the interlayer shear strength can also be given by

Circular mesa $$\tau_s = \frac{4\Gamma}{\pi D}$$

Square mesa $$\tau_s = \frac{\Gamma}{w}$$

As it can be seen, the interlayer shear strength is inversely proportional to the diameter (width) of the circular (square) mesas, implying that the smaller mesas can unexpectedly sustain greater shear stresses.

Supplementary Note 2.2. Source of uncertainties in IAE measurements

Regarding the uncertainties in our AFM experiments, the accuracy of force-displacement measurements can be limited mostly by the uncertainty in the determination of the interfacial contact area and spring constant of the AFM probe. The first source of uncertainty in the interfacial adhesion measurements is the surface area of the tip, which must be known to determine the interfacial adhesion energy. In order to create a known contact area, we used 2D crystal tips with a very well-defined geometric shape parallel to the substrate, enabling an atomically clean and flat contact interface. Our interfacial adhesion measurements reveal that the tilting angle between the tip and the substrate is smaller than 1°, indicating perfect face-to-face contact during measurements (**Supplementary Note 2.3**). We reduced the second main source of uncertainty in our measurements by determining the stiffness of the AFM cantilever by means of three different methods and took their mean value as the static normal (3.05±0.05 Nm^{-1}) and axial (8.60±0.40 Nm^{-1}) spring constants of the probe, suggesting a relative calibration error of 2% and 5%, respectively (**Supplementary Note 2.4**). Although a calibrated AFM probe was used for these experiments, the spring constant value still contains approximately ∼2-5% error. Thus, the same tip was used throughout all experiments to ensure that the relative change in the interfacial adhesion energy is accurate even though the absolute value may contain some systematic error. The laser spot was also kept at the same position on the lever to avoid any changes in the force measurements. After performing all the measurements, the spring constant of the probe was again determined in ambient conditions to make sure that the cooling/local annealing of the substrate has no appreciable effect on its stiffness, yielding the spring constant still within the uncertainty range of our measurements. We also note that the random crystalline orientation at the interface of 2D crystal tips and 2D crystal substrates has no appreciable effect on the IAE measurements (**Supplementary Note 2.5**).

Supplementary Note 2.3. Effect of tilt angle on IAE measurements

Flat tips can result in direct and accurate acquisition of the interfacial adhesion energy, provided they are carefully aligned with respect to the sample surface. In this study, an in-situ flattened tip was used as a means to minimize the effect of the tilt angle of the substrate on the IAE measurements. Nevertheless, to investigate the dependence of the IAE on the misalignment of the sample, we used a home-made setup consisting of a variable tilt mount with an angle resolution of 0.5° to measure the IAE of G tip on the G substrate at a contact pressure of 1 MPa and 10 MPa. The G substrate was first rotated about the axis perpendicular to the probe length to achieve the maximum pull-off force, followed by the rotation of the substrate about the axis parallel to the probe length with an increment of 0.5° for the subsequent IAE measurements at the G/G interface. Ten IAE measurements were taken from different locations of the G sample for each tilt angle. Given that the maximum IAE value is presumed for the fully aligned tip-sample system, we set the corresponding tilt angle to be zero and accordingly the IAE values are reported for tilt angles ranging from -3° to 3°, as shown in **Supplementary Fig. 8a**. It is seen that the IAE of G/G is almost independent of the contact pressure within the studied tilt angle range. It is also observed that the IAE exhibits a very weak dependence on the tilt angle of less than 1.5° (within our experimental errors) and only ~30% reduction at the tilt angle of 3°. This result is interesting because in the literature a larger adhesion force reduction of ~30% and 60% was reported at a tilt angle of 1° and 3°, respectively, for a flat silicon tip (nominal spring constant: 48 Nm^{-1}) with a 2 μm diameter contact area over a 2 nm thick SiO_x substrate [2]. Much smaller contact area in the present study (i.e., 60 nm versus 2 μm diameter flat tip apex) motivates us to investigate to what extent the misalignment effect on the interfacial adhesion force is controlled by the size of the contact area. To do so, we perform a set of classical MD simulations to calculate the pull-off force between the SiO_x substrate and the square flakes of graphene with both small (2 nm × 2 nm) and large (10 nm × 10 nm) flake sizes. The SiO_x substrate was tilted clockwise in the range of 0° to 3° and the pull-off force was obtained as the tapered silicon layer was moved away from the sample surface. For comparison purposes, the pull-off force was normalized with respect to the maximum pull-off force at a tilt angle of 0°. It is evident from **Supplementary Fig. 8b** that the pull-off force in the larger flakes exhibits a significantly stronger dependence on the tilt angle compared to that in the smaller flakes, further supporting our experimental observations that the smaller flat tip apex provides more reliable IAE values on the tilted substrates.

We also note that over an order of magnitude smaller torsional stiffness of our probe compared to that in [2] can play a role in the formation of conformal contact at the tilted G/G interface, leading to the lower sensitivity of our IAE measurements toward the tilt angle (according to the Euler-Bernoulli equation, the torsional stiffness $k_φ$ is proportional to the normal stiffness k_n of the probe of length L and can be given by: $k_φ=4k_nGL^2/(3E)$ where E and G are the Young's and shear moduli of silicon, respectively).

(a)

(b)

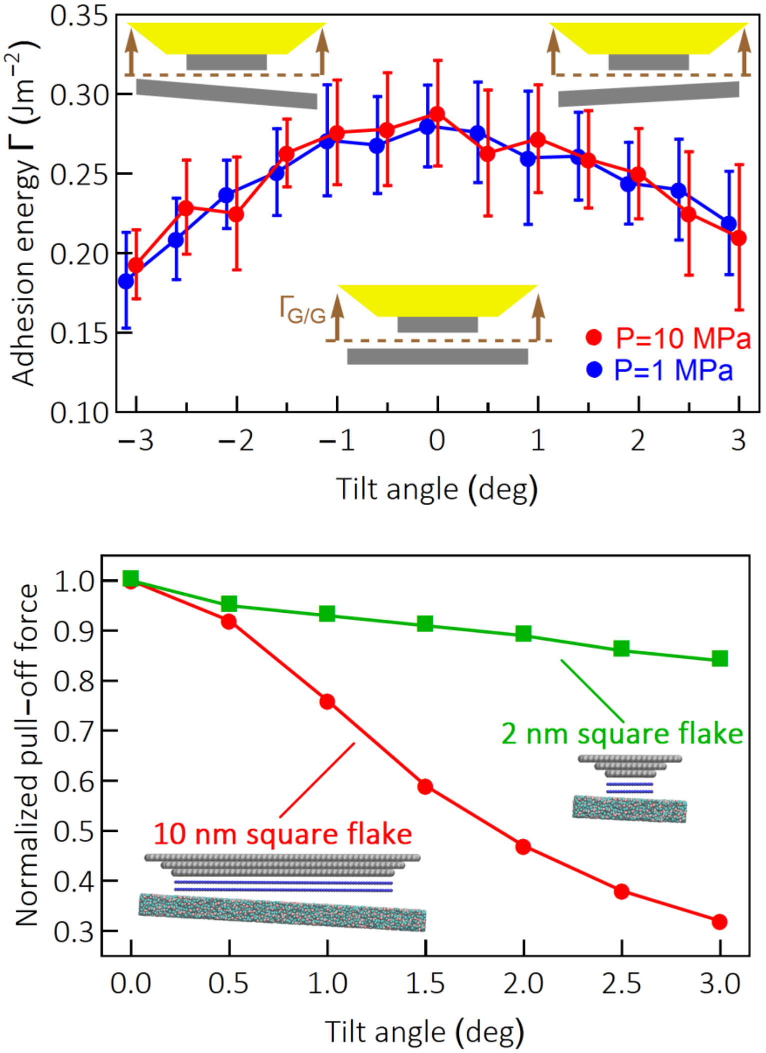

Supplementary Figure 8. Effect of tilt angle on IAE measurements. (a) Interfacial adhesion energy measurements of G/G as a function of tilt angle. (b) MD calculations of normalized pull-off force versus the tilt angle of the SiO_x substrate at the interface of SiO_x and the small (2 nm × 2 nm) and large (10 nm × 10 nm) graphene flakes.

Supplementary Note 2.4. Static spring constant calibration

The main source of uncertainty in our AFM measurements lies in the determination of spring constant k of the AFM probe subjected to the normal or shear force at the apex. A large number of studies have addressed this issue in the past and suggested different techniques producing more or less uncertainty in the calculation of this parameter. The method that is most viable for *in situ* characterization is the thermal method (independent of material properties of the probe), requiring knowledge of instrument parameters and mean-square amplitude vibrations of the cantilever as a function of frequency only, which can be efficiently and quickly measured before each experiment.

We measure thermal fluctuations of the free end of the cantilever to determine its spring constant, which, according to the equipartition theorem, is $= k_B T/\langle z^2(f)\rangle$, where T is the room temperature, k_B is Boltzmann's constant, and $\langle z^2(f)\rangle$ is the mean square of the cantilever thermal fluctuation amplitude. Because the thermal noise method relies on the measurement of cantilever fluctuations, some corrections are required to achieve an accurate determination of the spring constant.

Supplementary Note 2.4.1. Spring constant calibration under a normal load at the apex

(a) *Contribution of fundamental natural frequency*: Basically, $\langle z^2(f)\rangle$ is obtained by integrating the amplitude power spectrum of the cantilever (which is modeled by a simple harmonic thermally-driven oscillator) over the whole frequency range. However, in practice, the dominant contributions of the thermally driven fluctuations is at and around the fundamental natural frequency, f_1, and, as a result, the amplitude spectral density is recorded and integrated over a few *k*Hz band of frequencies centered at the fundamental natural frequency. Hence, the expression for the spring constant needs to be modified by

$$k = \frac{12}{\alpha_1^4}\frac{Q}{\pi}\frac{k_B T}{\langle z^2(f_1)\rangle}\frac{\Delta f}{f_1} \tag{3}$$

where $\alpha_1 = 1.8751$ is the parameter quantifying the amount of energy stored in the fundamental vibration mode, Q and $\langle z^2(f_1)\rangle$ denote quality factor and mean square cantilever displacement in the fundamental mode, respectively, and Δf is frequency resolution. An accurate measurement of mean square displacement in a region around the fundamental natural frequency is challenging for AFM probes and the following effects must be taken into account.

(b) *Conversion of virtual to actual deflection*: The AFM measures the angular changes (virtual deflection, $\hat{z}$) rather than the actual deflection, z, so it is necessary to correct the mean-square amplitude of the cantilever vibration to account for these angular changes. The actual deflection z for the fundamental mode can be related to the virtual deflection $\hat{z}$ through the following expression [3]

$$\langle z^2(f_1)\rangle = \left(\frac{3}{2}\frac{\sin\alpha_1 + \sinh\alpha_1}{\alpha_1 \sin\alpha_1 \sinh\alpha_1}\right)^2 \langle \hat{z}^2(f_1)\rangle \tag{4}$$

(c) *Effect of the protruding tip*: In the contact mode, the adhesion force is applied to the tip apex not to the free end of the cantilever. Hence, we need to take the offset end load into consideration. As schematically illustrated in **Supplementary Fig. 9a**, a tip protruded from the free end of a cantilever tilted by an angle θ with respect to a horizontal surface increases the effective lever of the force from $L\cos\theta - x$ to $L\cos\theta + D\sin\beta - x$, where the effective tip height D is measured from the tip apex to the midpoint of the cantilever thickness.

Following the work of Hutter [4], the bending moment-curvature relation in the cantilever can be expressed by the following differential equation

$$EI\cos^3\theta \frac{d^2 z}{dx^2} = F(L\cos\theta + D\sin\beta - x) \tag{5}$$

After integrating and applying the boundary condition $dz/dx\,(x=0) = -\tan\theta$, we can find the inclination at the free end about the equilibrium slope as follows

$$\Delta\hat{z} = \frac{dz}{dx} = \frac{FL}{2EI\cos^2\theta}(L\cos\theta + 2D\sin\beta) \tag{6}$$

Further integration of Eq. (5) and using the boundary condition $z(x=0) = 0$ lead to the deflection at the free end of the cantilever as follows

$$\Delta z = \frac{FL^2}{3EI}\left(L\cos\theta + \frac{3}{2}D\sin\beta\right)\cos\theta \tag{7}$$

By inspection of Eqs. (6) and (7), the relation between the actual and virtual deflections in Eq. (4) is further modified to

$$\langle z^2(f_1)\rangle = \left(\frac{3}{2}\frac{\sin\alpha_1 + \sinh\alpha_1}{\alpha_1 \sin\alpha_1 \sinh\alpha_1}\right)^2 \left(\frac{2L\cos\theta + 4D\sin\beta}{2L\cos\theta + 3D\sin\beta}\right)^2 \langle \hat{z}^2(f_1)\rangle \tag{8}$$

(d) *Effect of size and position of the laser spot*: The laser spot size is primarily defined by the laser wavelength and microscope objective being used. While the minimum achievable spot size is diffraction limited, according to the laws of physics and optics, the laser spot diameter D_s is equal to 1.22 λ / N.A., where λ is the wavelength of the laser, and N.A. is the numerical aperture of the microscope objective being used. In our AFM setup with an 830 nm laser and a 0.28/10x objective, the theoretical spot diameter is 3.62 μm. Such a small optical spot (compared to the long length of the probe, i.e., D_s/L_{eff} = 3.62 μm/(231.4 μm×cos12º) = 0.016) which is placed at the very end of the cantilever has a negligible effect on the correction factor considered in Eq. (4) [5].

(e) *Effect of the angle of repose*: The optical lever sensitivity, S, defined by the ratio of the displacement of the piezo-scanner Δz_0 and the position-sensitive photo-detector (PSPD) voltage ΔV_0, is the factor that allows us to convert the cantilever deflection from volts to nanometers. Basically, the optical lever sensitivity is the slope of a force curve obtained by placing the probe in contact with an infinitely stiff surface and ramping the scanner position. However, the value of S (=$\Delta z_0/\Delta V_0$) is obtained in the scanner's reference frame while the contact force acts in the cantilever's reference frame, which is tilted at a repose angle of θ with respect to the sample. Hence, we need to divide S by $\cos\theta$, leading to

$$\langle z^2(f_1)\rangle = \left(\frac{3}{2}\frac{\sin\alpha_1 + \sinh\alpha_1}{\alpha_1 \sin\alpha_1 \sinh\alpha_1}\right)^2 \left(\frac{2L\cos\theta + 4D\sin\beta}{2L\cos\theta + 3D\sin\beta}\right)^2 \left(\frac{S}{\cos\theta}\right)^2 \langle \Delta V^2(f_1)\rangle \qquad (9)$$

where $\langle \Delta V^2(f_1)\rangle$ is the mean square voltage at the fundamental natural frequency.
Substituting Eq. (9) into (3), we end up with a full expression for the spring constant of the probe as follows

$$k = \frac{16}{3\alpha_1^2}\left(\frac{\sin\alpha_1 \sinh\alpha_1}{\sin\alpha_1 + \sinh\alpha_1}\right)^2 \left(\frac{2L\cos\theta + 3D\sin\beta}{2L\cos\theta + 4D\sin\beta}\right)^2 (\cos^2\theta)\frac{Qk_BT}{\pi S^2\langle \Delta V^2(f_1)\rangle}\frac{\Delta f}{f_1} \qquad (10)$$

By setting $\alpha_1 = 1.8751$, Eq. (10) can be further simplified to

$$k = 0.8174\left(\frac{2L\cos\theta + 3D\sin\beta}{2L\cos\theta + 4D\sin\beta}\right)^2 (\cos^2\theta)\frac{Qk_BT}{\pi S^2\langle \Delta V^2(f_1)\rangle}\frac{\Delta f}{f_1} \qquad (11)$$

Our XE-70 AFM head provides a tilt angle of $\theta = 12°$. From the SEM measurements of the probe geometry, the following parameters were determined: $\beta = 10.5°$, $L = 231.4\mu m$, $D = 20.7\mu m$. All measurements were performed in the ambient conditions at $T = 295\,K$. Three parameters Q, f_1, and $\langle \Delta V^2(f_1)\rangle$ are determined by acquiring the thermal noise power spectrum using the SR760 FFT Spectrum Analyzer with the frequency resolution of $\Delta f = 15.625$ Hz. The optical lever sensitivity, S, on the sapphire sample was 14.32 nm/V.

Supplementary Fig. 9b shows the typical measurement of the mean square voltage amplitude by connecting the spectrum analyzer to the output of the preamplifier of the PSPD of the AFM controller. The spectrum analyzer measures the mean square voltage amplitude for the fundamental cantilever oscillation mode, which can be represented as

$$\langle \Delta V^2(f)\rangle = \frac{A}{f} + B + \frac{\langle \Delta V^2(f_1)\rangle}{Q^2}\frac{1}{[1-(f/f_1)^2]^2 + (f/Qf_1)^2} \qquad (12)$$

where the first two terms are used to account for a 1/f noise background and a white-noise floor, respectively. Once Eq. (12) is fitted to the data by five fit parameters A, B, Q, f_1, and $\langle \Delta V^2(f_1)\rangle$, the last three ones are used for the calculation of the spring constant from Eq. (11) which is obtained to be 3.05 Nm^{-1}. In a second approach, we employed the Sader method [6] with the input parameters of $(L, w, f_1, Q) = (230.8\mu\text{m}, 36.4\mu\text{m}, 73.375\text{kHz}, 225.1)$, yielding a static spring constant of 3.29 Nm^{-1}. This is slightly higher than the value predicted by Eq. (11) because the effects of the protruding tip and the angle of repose were not taken into account. After considering these effects, the spring constant becomes 3.10 Nm^{-1}, more consistent with that of thermal noise method. Note that the effective length in the Sader method was modified to account for the triangular end of the cantilever such that the new rectangular cantilever exhibits the exact same deflection response as the original cantilever. In a third approach, we estimated the spring constant of the probe by performing a finite element method simulation using COMSOL™. The effective dimensions of the probe were measured using SEM images to an accuracy of ±0.5 μm and ±2 μm for the width and length, respectively, and ±100 nm for the thickness. Assuming that the probes are typically fabricated

from a [100] wafer with their long axis aligned with a <110> direction, we considered the elastic modulus of silicon to be E_{110}=169 GPa. After tilting the probe by 12° about the x axis, we applied a 30 nN force in the positive z direction to the tip apex while the root of the cantilever was fixed (**Supplementary Fig. 9c**). From the computed deflection, the stiffness of the probe was calculated to be 2.99 Nm^{-1}, in excellent agreement with the static spring constant obtained from thermal noise and Sader methods. For the purpose of this experiment, the mean value of the three methods (3.05±0.05 Nm^{-1}) was taken as the static normal spring constant, enabling us to obtain the adhesion force by $F = kS\Delta V$, where ΔV is the measured PSPD voltage signal.

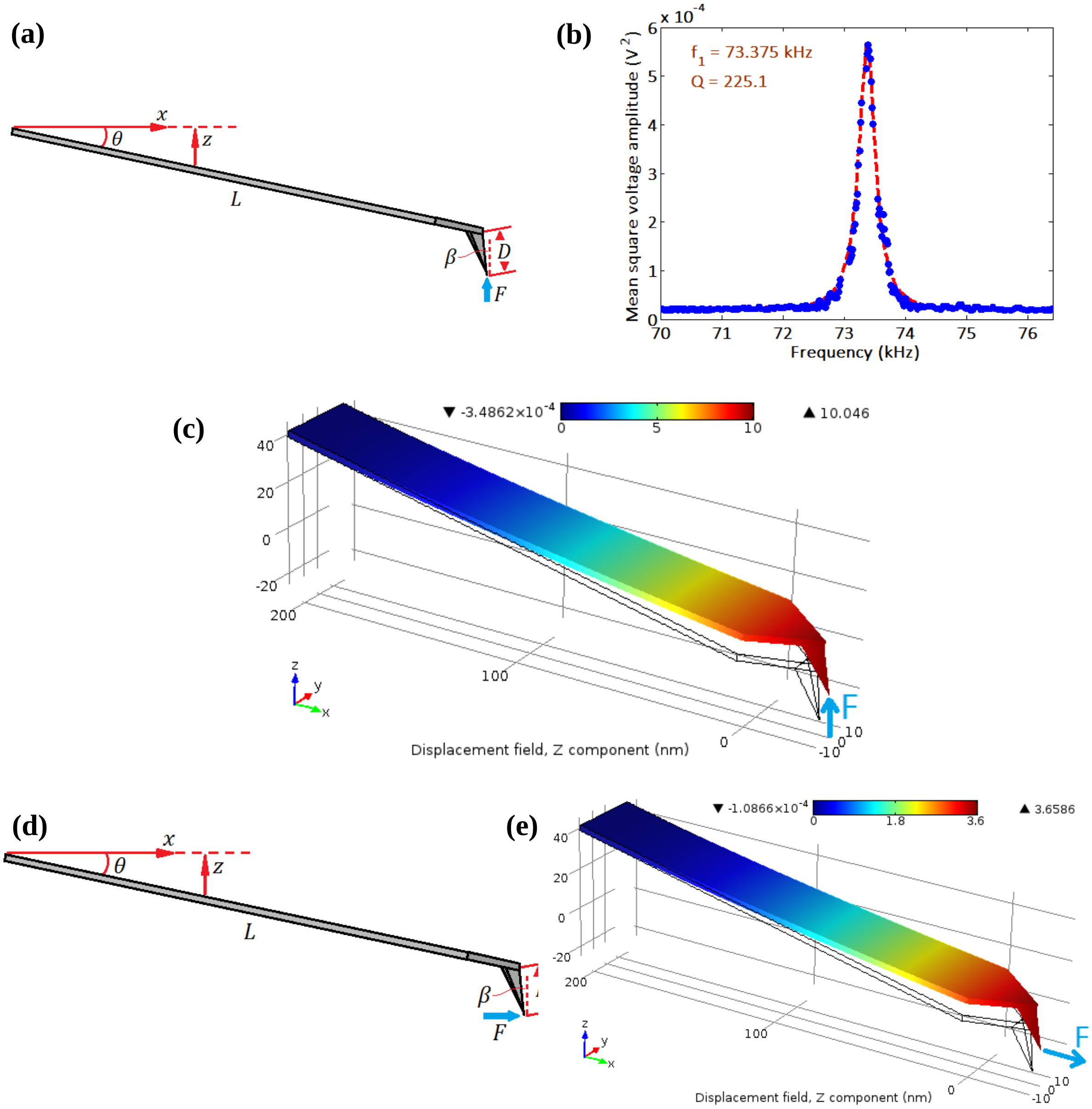

Supplementary Figure 9. Spring constant calibration of AFM probe. (a) Schematic drawing of a cantilever tilted by an angle θ with respect to a horizontal surface subjected to a normal force along the positive z axis. (b) Mean square fluctuations in amplitude as a function of frequency for the probe with the protruded tip in air. The blue curve is the original data while the dash curve is the fit to the data using Eq. (12). (c) Deflection of the probe when a contact force of 30 nN is applied to the apex in the positive z direction, yielding a spring constant of 2.99 Nm^{-1}. (d) Schematic drawing of a cantilever tilted by an angle θ with respect to a horizontal surface subjected to an axial force directed to the long axis of the cantilever along the positive x axis. (e) Deflection of the probe when a contact force of 30 nN is applied to the apex in the positive x direction, yielding a spring constant of 8.20 Nm^{-1}.

Supplementary Note 2.4.2. Spring constant calibration under an axial load at the apex

In this study, we moved 2D crystal substrates along the long axis of the cantilever tip rather than perpendicular to its long axis to obtain more accurate shear force measurements. In order to demonstrate the higher force resolution in the present axial shear force microscopy technique compared to that in the conventional lateral shear force microscopy technique, we determine the spring constant of the probe for each case.

The spring constant of the probe under the axial load can be related to the previously calibrated spring constant under the normal load by calculating the deflection ratio of the free end of the cantilever under normal and axial loading conditions. In the contact mode, the axial force directed to the long axis of the cantilever is applied to the tip apex, as shown in **Supplementary Fig. 9d**. The bending moment-curvature relation in the cantilever can be expressed by the following differential equation

$$EI\cos^3\theta\,\frac{d^2z}{dx^2} = F(L\cos\theta - x)\tan\theta + FD\cos\beta \qquad (13)$$

After integrating Eq. (13) twice with respect to x and applying the relevant boundary conditions, we can find the deflection at the free end of the cantilever as follows

$$\Delta z = \frac{FL^2}{3EI}\left(L\sin\theta + \frac{3}{2}D\cos\beta\right)\cos\theta \qquad (14)$$

By inspection of Eqs. (7) and (14), one can analytically determine the deflection ratio for axial and normal loads by

$$\frac{\Delta z_{\mathrm{axial}}}{\Delta z_{\mathrm{normal}}} = \frac{2L\sin\theta + 3D\cos\beta}{2L\cos\theta + 3D\sin\beta} = 0.339 \qquad (15)$$

Because the spring constant is inversely proportional to the tip deflection, the spring constant of the probe under the axial load can be determined by (3.05 Nm^{-1})/0.339 = 9.00 Nm^{-1}. In a second approach, we carried out a similar finite element method simulation with the axial load of 30 nN applied to the positive x direction (**Supplementary Fig. 9e**). From the computed deflection, the spring constant of the probe was calculated to be 8.20 Nm^{-1}. For the purpose of this experiment, the mean value of the two methods (8.60±0.40 Nm^{-1}) was taken as the static spring constant under the axial load. This value is an order of magnitude smaller than the lateral spring constant of the probe (i.e., 83.8 Nm^{-1} when the shear force acts perpendicular to the long axis of the cantilever), indicating much more accurate shear force measurements in the present shear force microscopy technique. This is also confirmed by our shear force measurements with subnano-level resolution (**Fig. 1c** in the main text).

Supplementary Note 2.5. Effect of crystalline orientation on IAE measurements

Owing to the random crystalline orientation at the interface of 2D crystal tips and 2D crystal substrates, the effect of such interlayer lattice mismatch on the IAE measurements should be investigated. A comparison between our standard deviations of the measured interfacial adhesion energy at intact G/G, hBN/hBN and MoS_2/MoS_2 interfaces (at which we expect perfect AB stacking with no contamination) and those at the pre-cooling treated G/G, hBN/hBN and MoS_2/MoS_2 interfaces (at which we believe there could be interlayer lattice mismatch with small amount of contamination) reveals that the relative orientation of 2D vdW crystals may contribute relatively little to their overall interfacial adhesion energy. The average of the standard deviations of the interfacial adhesion energy at the intact and the pre-cooling treated interfaces is ±0.027 and ±0.029 (G/G), ±0.026 and ±0.033 (hBN/hBN) and ±0.033 and ±0.037 (MoS_2/MoS_2), respectively, indicating 7%, 27% and 12% increase, respectively, in the standard deviations due to the effect of both interlayer lattice misorientation and the interfacial contamination. This is also confirmed by the first direct experimental measurement of the interfacial adhesion energy of the G crystal using the micro-force sensing probe, showing that the interlayer twist angle ranging from 0° (perfect AB stacking) to 54° has only a weak effect (about 5.4%) on the interfacial adhesion energy [7]. Moreover, the critical adhesion forces of G-wrapped AFM tip on G, hBN and MoS_2 substrates reveal that the random crystalline orientation has negligible effect on the interfacial adhesion force measurements whose very small standard deviations of about 1% are closely related to the instability point at which the tip is pulled off from the sample surface [8].

Supplementary Note 2.6. Possible edge functionalization of 2D crystals

Among all our IAE measurements at 2D crystal/2D crystal and 2D crystal/SiO_x interfaces, only ~ 5% of *F-d* curves at the G/SiO_x interface exhibit the possible short-range bond at the edge of the G nanomesa. Although 2D crystals possess intrinsic active edge sites, the etching process of nanomesas could also functionalize or chemically modify their edge. Gongyang et al. [52] used a micro-force sensing probe to study the effect of chemical bonds (induced by the reactive ion etching at the edge of 4 μm×4 μm graphite mesas) on the cohesion energy of G/G through a direct shear force technique. They showed that the cohesion energy in the presence of chemical bonds at the edge (~ 4.80 Jm^{-2}) is an order of magnitude larger than that in the absence of chemical/physical bonds (~ 0.37 Jm^{-2}). From **Figs. 1c** and **1e** in the main text, the cohesion energy of G crystal obtained by our shear force measurements is consistently less than 0.361±0.014 Jm^{-2}, confirming no chemical bond/dangling bond both at the edge and along the sliding plane of G nanomesas. Similarly, the level of cohesion energy in hBN and MoS_2 crystals dictates no chemical bond at their edge. Moreover, Gongyang et al. [52] showed that the effect of chemical bonds on the shear force can be eliminated by annealing the G micromesas at 150 °C. This, coupled with the fact that our measured cohesion energy at the intact homointerfaces is independent of the annealing temperatures (gray circles in **Fig. 2a**) further confirms that the chemical bonds near/at the edge of nanomesas have no appreciable effect on our measurements even at room temperature. As such, we believe that at least the edge of the most bottom layer of the tip-attached nanomesas (where the sliding/separation takes place) is unlikely to be functionalized due to the etching process, and, therefore, the edge functionalization has no contribution to the overall IAE measurements. Moreover, the observation of chemical bonds at the edge of G nanomesa in some *F-d* curves of G/SiO_x heterostructures can be attributed to the intrinsic active edge sites in the most bottom layer of the G crystal tip rather than any possible edge functionalization due to the etching process of G nanomesas.

Supplementary Note 3. Surface roughness measurements

To gain a sub-nanoscale insight into the origin of the distinctive interfacial behavior in the G/SiO_x heterostructure specifically and into the underlying interaction mechanism of 2D crystals and SiO_x in general, the interfacial contact of 2D crystal nanomesas with SiO_x substrate alone does not provide a direct access to the 2D crystal/SiO_x interface. Therefore, three-dimensional surface topography measurements of single layer 2D crystals on SiO_x with sub-nanometer resolution together with the power spectral density (PSD) analysis of the surface roughness data provides a versatile means to explore the interfacial behavior of 2D crystal/SiO_x heterostructure. To do so, 2D crystal stamps with 10 μm square mesas of thickness 10-30 nm are mechanically transferred onto a flat polydimethylsiloxane (PDMS) substrate using a combined imprint-assisted shear exfoliation and transfer printing technique [9]. The flat PDMS stamp with the uniform exfoliated multilayer mesas enables a fully conformal contact with the SiO_x substrate under a uniform pressure. A lab-made roller tool [10] was used to transfer the 2D crystal mesas from the PDMS substrate to the 90 nm thick SiO_x/Si substrate at a contact pressure of 5 MPa. Prior to the flake transfer, SiO_x samples were sonicated in acetone, isopropanol and deionized water and dry blown with nitrogen, followed by annealing at 200 °C. Raman spectroscopy coupled with AFM height profile measurements were used to determine the layer number of the exfoliated mesas with monolayer accuracy.

The most popular parameter characterizing the morphology of surfaces is the root mean square (RMS) roughness, which describes the RMS height of an $L \times L$ scanned surface area around its mean value as follows

$$R_q = \sqrt{\frac{1}{N^2}\sum_{m=1}^{N}\sum_{n=1}^{N} Z^2(x_m, y_n)} \tag{16}$$

where N represents the number of grid points in *x*- or *y*-direction with a pixel size of $\Delta L \times \Delta L$ ($\Delta L = L/N$), $Z(x_m, y_n)$ is the height of the surface relative to the mean line at position (x_m, y_n), and $x_m = m\Delta L$, $y_n = n\Delta L$. Despite its reliable information on the height deviation, the RMS surface roughness can neither distinguish between peaks and valleys nor describe the lateral distribution of surface features. Using a fast Fourier transform (FFT) algorithm, the power spectral density (PSD) can however provide a more accurate and comprehensive description of

the surface roughness both in vertical and lateral directions. To analyze the AFM image data, we adopt the following two-dimensional (2D) PSD function

$$\mathrm{PSD}(f_x, f_y) = \frac{1}{L^2}\left\{\sum_{m=1}^{N}\sum_{n=1}^{N} Z(x_m, y_n)\exp\left[-2\pi i\Delta L\left(mf_x + nf_y\right)\right](\Delta L)^2\right\}^2 \tag{17}$$

where f_x and f_y are the spatial frequency in the *x*- and *y*-directions, respectively, which take the discrete range of values $1/L$, $2/L$, . . . , $N/2L$. According to the Parseval's theorem, the square root of the area under the PSD curve is equal to the RMS roughness.

Using a 16-bit digital-to-analog converter in low voltage mode with an ultralow noise AFM controller can significantly improve the X-Y scanner's lateral resolution to subnanometer ($L/2^{16}$) and the Z scanner's vertical resolution to sub-angstrom (at the expense of limiting the X-Y and Z scanners' motion range), allowing us to image atomic-scale features. Given that the radius of the AFM tip determines the maximum spatial frequency (i.e., $N/2L$) that can be measured, 200 nm square images with 256 × 256 pixel resolution were captured for each 2D material flake at a scanning rate of 0.5 Hz, providing a pixel size of 0.8 nm smaller than the probe tip radius of <2-5 nm. To obtain a more accurate PSD of the surface, at least twenty data sets obtained from different locations of the sample were processed and then averaged for each case. A first-order regression polynomial was selected to remove any artifacts that result from the slope (consistently less than 0.1°) produced by the scanning process. Since the features smaller than 2 nm (i.e., spatial frequency > 0.5 nm^{-1}) may not be captured in our setup due to contributions from both the limited size of the tip radius and instrumental noise, we applied a low pass filter to the 2D PSD of the AFM topographic images to suppress <2 nm features without any effect on the physical content of the image data [11, 12].

In order to perform accurate and repeatable surface roughness measurements, the tip sharpness and the system noise floor play a key role. We used an ultrasharp tip with 2 nm nominal radius of curvature (<5 nm guaranteed) and sparing constant of 39.1 Nm^{-1} in the noncontact mode and in the attractive regime (with a frequency shift of -10 Hz and free amplitude of 7.5 nm) under ambient conditions and then determined the noise floor of the AFM system (being consistently less than 0.3 Å) by measuring the average surface roughness under the following conditions: contact mode with 256 × 256 pixel resolution and zero scan size (i.e., the tip apex remains in static contact with the sample surface at a single point). To ensure the tip sharpness is preserved throughout the roughness measurements, we performed more than 50 sequential imaging with sub-angstrom precision in non-contact mode of the same area on the SiO_x surface and monitored the variation of the surface roughness measurements. We acquired an average surface roughness value of 310 pm with a standard deviation of 5 pm.

Supplementary Note 4. X-ray photoelectron spectroscopy on pre-annealed MoS_2 crystals

Supplementary Fig. 10a shows x-ray photoelectron spectroscopy (XPS) measurements on both freshly exfoliated and pre-annealed MoS_2 samples at excitation energy of 1486.6 eV. Residual electrostatic charging effects were taken into account by applying an offset to the spectra with a reference signal of C (1*s*) at a binding energy of 284.6 eV. In order to eliminate any interference between the dominant Mo (3*d*) and S (2*s*) features, we used the less intense Mo (3*p*) photoelectron signal for the quantification purposes. Each set of peaks was fitted by a 70% Gaussian-30% Lorentzian function. In addition, peaks of spin-orbit doublets Mo ($3p_{3/2}$ and $3p_{1/2}$) were set to have an area ratio in accordance with quantum degeneracy values (i.e., 2:1 for $3p_{3/2}$ and $3p_{1/2}$ orbitals).

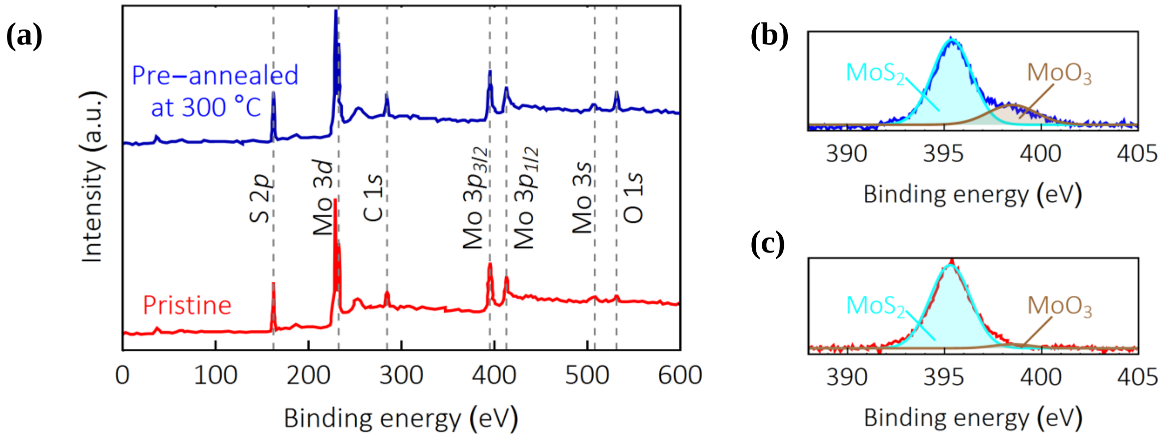

Supplementary Figure 10. X-ray photoelectron spectroscopy of MoS_2 crystal. (a) The survey XPS spectra of pristine and pre-annealed MoS_2 crystal at 300 °C. Comparison between high-resolution Mo (3$p_{3/2}$) XPS spectrum of (b) pre-annealed MoS_2 crystal at 300 °C and (c) pristine MoS_2 crystal.

It is evident from **Supplementary Fig. 10a** that the pre-annealed MoS_2 at 300 ºC exhibits a higher intensity of oxygen species on the surface as compared to the freshly exfoliated MoS_2 sample. More inspections of the deconvoluted photoelectron signal for Mo (3$p_{3/2}$) in **Supplementary Figs. 10b** and **10c** confirm this observation, yielding a MoS_2/MoO_3 ratio of 4.5 for the pre-annealed samples at 300 ºC.

Supplementary Note 5. Comparative studies of IAE

In this section, we perform a comprehensive comparison study on the interfacial adhesion energy (IAE) of 2D crystals and 2D crystal/SiO_x heterostructures obtained from a wide range of experimental methods. While a vast majority of studies have been conducted on the interaction of G with G (**Supplementary Note 5.1**) and SiO_x (**Supplementary Note 5.2**) substrates with a wide range of reported IAE values, to the best of our knowledge, no IAE measurement at the hBN/hBN and hBN/SiO_x interfaces yet exists, and also there are a very limited number of reports on the interaction of MoS_2 with MoS_2 (**Supplementary Note 5.1**) and SiO_x (**Supplementary Note 5.2**) substrates. We also note that, to the best of our knowledge, there is no direct IAE measurement on the 2D crystal heterostructures.

Supplementary Table 1. Cohesion energy of carbon nanotubes, few-layer graphene, and graphite.

Method	Sample	Stack	Γ (Jm^{-2})	Ref
Heat of wetting	Graphite	N.A.	0.26±0.03	[21]
Radial deformation of MWCNT	Collapsed MWCNT	(Non–)AB*	0.15–0.31	[22]
Thermal desorption	HOPG	AB	0.37±0.03	[23]
MWCNT retraction	MWCNT	Non–AB	0.28–0.4	[24]
Deformation of thin sheets	HOPG	AB	0.19±0.01	[25]
AFM pull-off force measurements	HOPG	Non–AB	0.319±0.05	[26]
DWCNT inner-shell pull-out	DWCNT	Non–AB	0.436±0.074	[27]
SEM peeling of MWCNT	Collapsed MWCNT on 1-LG	(Non–)AB	0.40±0.18	[28]
	Flattened MWCNT on 1-LG		0.72±0.32	
AFM-assisted mechanical shearing	HOPG	Non–AB	0.227±0.005	[15]
Self-retraction motion	HOPG	AB	0.39±0.02	[13]
		Non–AB	0.37±0.01	
AFM-assisted mechanical shearing	HOPG	Non–AB	0.35	[14]
AFM nano-indentation	BLG/FLG onto FLG	Non–AB	0.307±0.041	[29]
Atomic intercalation of neon ion	1LG onto HOPG	Non–AB	0.221±0.095	[30]
Surface force balance	CVD-grown 1LG/1LG	Non–AB	0.230±0.008	[31]
	CVD-grown FLG/FLG	Non–AB	0.238±0.006	

* Intermediate between commensurate and incommensurate states

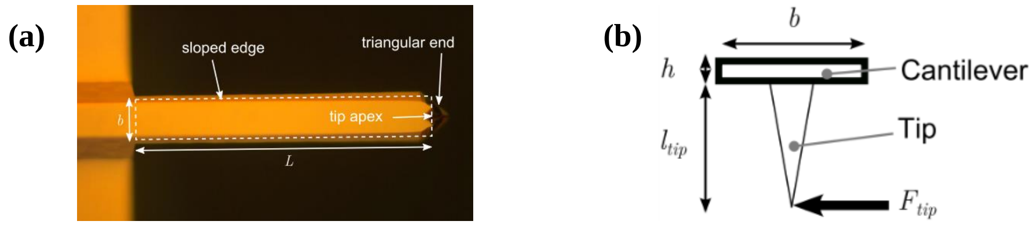

(c)

Cantilever		Dimensions			
Tip	Experimental Structure	L (μm)	b (μm)	h (μm)	l_{tip} (μm)
A	50 nm radius mesa	217±5	30±1	2.9 ±0.1	12.2 ±0.1
B	100 nm, 200nm & 300nm radius mesas	218±5	30±1	2.9 ±0.1	11.5 ±0.1
C	150 nm & 200 nm radius mesas	218±5	30 ±1	2.8 ±0.1	11.7 ±0.1
D	Circle & Beam Structure	218±5	30±1	2.7 ±0.1	11.9 ±0.1

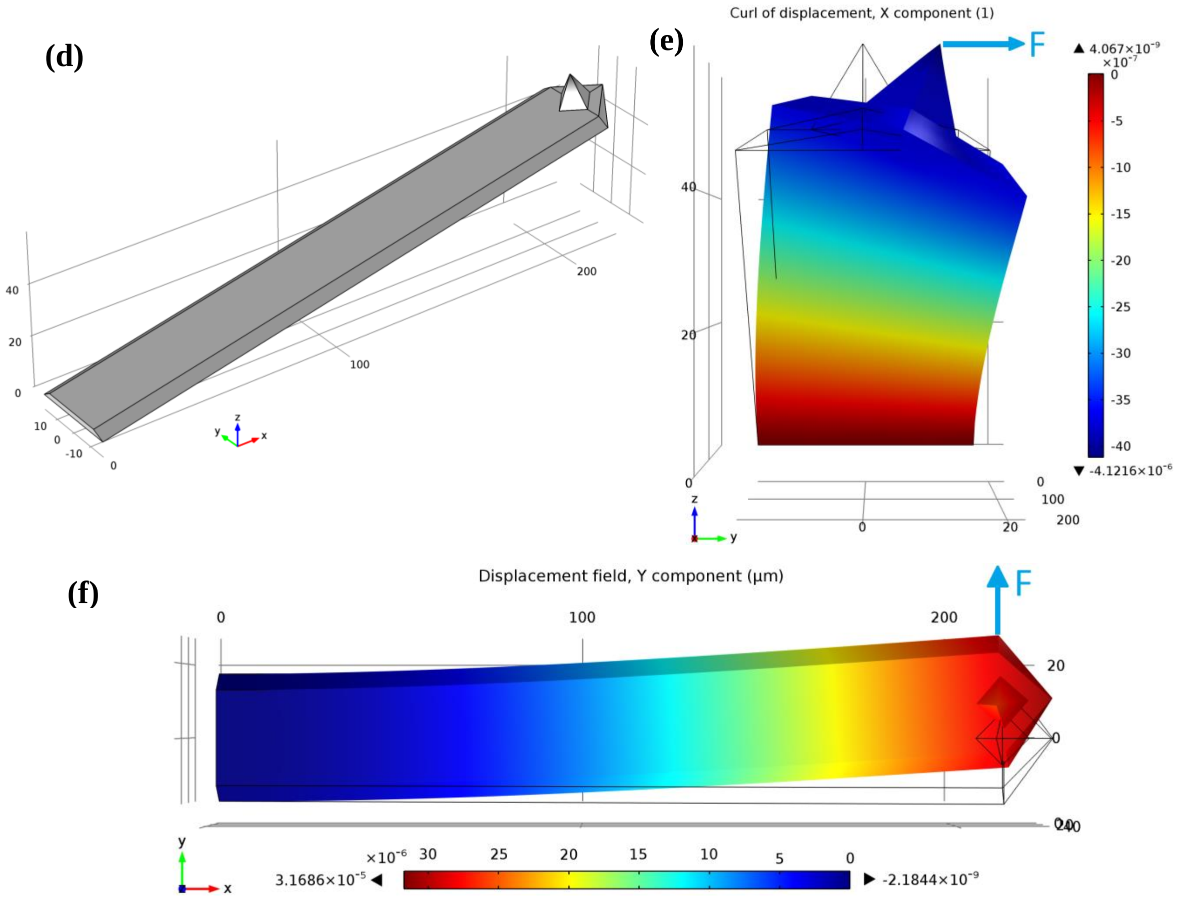

Supplementary Figure 11. Rotation and lateral deflection of AFM probe. (a) Typical optical microscope image of the AFM tip with an effective length and width of L and b, respectively. (b) Schematic drawing of probe dimensions and a lateral load applied to the apex. (c) Dimensions of the probes used. (a)-(c) are directly used from [15]. (d) Corresponding 3D model of the AFM probe. (e) Rotation of the probe A about its long axis x when a lateral force of 10 nN is applied to the tip apex in the positive y direction, yielding a torsional stiffness value of c_{φ}=222.74 Nm^{-1}. (f) Lateral deflection of the probe A when a force of 10 nN in the positive y direction is applied to a point on the cantilever shank with the same x distance from the cantilever root as the x distance of the tip apex, yielding a lateral bending stiffness value of k_l=344.59 Nm^{-1}.

Supplementary Note 5.1. Comparison study on cohesion energy of 2D crystal homostructures

Although many attempts have been made over the last six decades to measure the cohesion energy of G crystal with the reported values ranging from 0.15–0.72 Jm^{-2} (**Supplementary Table 1**), there are few direct measurements of cohesion energy available for comparison. We found out that our measurements for cohesion energy of G crystal are in excellent agreement with micro-force sensing probe measurements on 4 μm wide square mesas (0.37±0.01 Jm^{-2} [13]) and AFM-assisted shearing measurements on 3 μm wide square mesas (0.35 Jm^{-2} [14]), but inconsistent with recent AFM-assisted shearing measurements on circular mesas of 100–600 nm in diameter (0.227±0.005 Jm^{-2} [15]).

We revisited the lateral stiffness calibration of all probes used in ref. [15] by means of a 3D finite element simulation. A typical optical microscope of the probes (**Supplementary Fig. 11a**), schematic of the probe dimensions and the lateral load directed perpendicular to the long axis of the cantilever (**Supplementary Fig. 11b**) and also their corresponding measured dimensions (**Supplementary Fig. 11c**) were all given in the supplementary materials of ref. [15]. We also used the same Young's modulus of 169 GPa and shear modulus of 50.9 GPa as they did.

After carefully developing the 3D model of the probe in COMSOL based on the given optical image and dimensions (**Supplementary Fig. 11d**), we first applied a lateral force of F_{tip}=10 nN to the tip apex and calculated the rotation of each probe φ about its long axis at a point right above the tip apex on the cantilever shank (with the same x and y coordinates as those of the tip apex) where the laser spot is normally positioned (**Supplementary Fig. 11e**), followed by calculating the torsional stiffness $c_\varphi=k_\varphi/l_{\text{tip}}^2$ where $k_\varphi=(F_{\text{tip}}l_{\text{tip}}/\varphi)$. It is to be noted that l_{tip} should have been replaced by $l_{\text{tip}} + h$ (i.e., the vertical distance between the acting point of the force at the tip apex and the location of the laser spot) for the accurate calculation of c_φ and k_φ, however, for comparison purposes, we used the same equation as Koren et al. [15] did. We next applied a similar lateral force of 10 nN to the same point of laser incidence on the cantilever shank (**Supplementary Fig. 11f**), followed by calculating the lateral stiffness $k_l=(F_{\text{tip}}/\Delta y)$ where Δy is the lateral deflection at the point of applied force. We finally calculated the effective lateral stiffness of the probe as follows $c_l=(1/k_l + 1/c_\varphi)^{-1}$.

It is seen from **Supplementary Table 2** that our 3D model predicts consistently stiffer (~1.5 times) probes than those described in the original work. We also noted that the normal spring constant of all probes k_n obtained by our simulations (with an average value of 3.04 Nm^{-1}) is also more than twice the value of Koren et al. (1.40 Nm^{-1}) [15], further indicating a systematic underestimation in their stiffness values. Using the modified lateral spring constant of 132 Nm^{-1} yields a cohesion energy value of 0.340±0.008 Jm^{-2} at the G/G interface, which is more consistent with our measurements.

Using the similar finite element technique, we also calculated the effective lateral stiffness of our probe to be 83.8 Nm^{-1}, which is an order of magnitude larger than the axial spring constant of the probe (8.60±0.40 Nm^{-1}), confirming that the accuracy of shear force measurements using the conventional lateral shear force microscopy technique is highly limited by the large spring constant of the probe.

Supplementary Table 2. Comparisons between calibrated stiffness of probes reported by Koren et al. [15] **and the present work.**

**Type**	$k_n(\mathrm{Nm^{-1}})$		$c_\varphi(\mathrm{Nm^{-1}})$		$k_l(\mathrm{Nm^{-1}})$		$c_l(\mathrm{Nm^{-1}})$		**Ratio**
	Koren	Present	Koren	Present	Koren	Present	Koren	Present	
A	1.19	3.31	218	223	183	343	99	135	1.36
B	1.40	3.26	202	248	150	338	86	143	1.66
C	1.71	2.94	168	217	137	327	75	130	1.73
D	1.30	2.63	176	189	160	315	84	118	1.40
**Avg.**	**1.40**	**3.04**	**191**	**219**	**158**	**331**	**86**	**132**	**1.53**

Supplementary Note 5.2. Comparison study on IAE of 2D crystal/SiO_x

Despite many experimental studies devoted to the IAE determination of 2D crystals/SiO_x heterostructures, no experimental data are available on the interaction of hBN/SiO_x, whereas the reported IAE data on the interaction of

G and MoS_2 with SiO_x are very diverse, ranging from 0.09–0.90 Jm^{-2} at the G/SiO_x interface (**Supplementary Table 3**) and 0.08–0.48 Jm^{-2} at the MoS_2/SiO_x interface (**Supplementary Table 4**). We believe that a part of this large data scattering can be attributed to different surface properties of SiO_x during sample preparation, leading to different surface roughness, surface configurations (due to its amorphous nature), surface polarities, charge impurities, surface reactions with ambient humidity, and type of surface termination/defects (i.e., H–, Si– and O–terminated surfaces).

Supplementary Table 3. Interlayer adhesion energy of carbon nanotubes, few-layer graphene, and graphite on SiO_x.

Method	Sample	Γ (Jm^{-2})	Ref
AFM nano-indentation	BLG/FLG	0.270±0.020	[29]
Pressurized blister	1LG	0.45±0.02	[32]
	2-5LG	0.31±0.03	
Pressurized blister	1LG	0.24	[33]
Pressurized blister	1LG	0.140±0.040	[34]
	5LG	0.160±0.060	
AFM with a microsphere tip	1LG	0.46±0.02	[35]
Intercalation of nanoparticles	5LG	0.302±0.056*	[36]
Infrared crack opening Interferometry	1LG	0.357±0.016	[37]
Nanoparticle-loaded blister	1LG	0.453±0.006	[38]
	3-5LG	0.317±0.003	
	10-15LG	0.276±0.002	
Intercalation of nanoparticles	FLG	0.567	[39]
Colorimetry technique	2LG	0.9	[40]
Interfacial nanoblisters	1LG	0.093±0.001	[41]

* After making a correction in E from 0.5 to 1 TPa.

Supplementary Table 4. Interlayer adhesion energy of MoS_2 on SiO_x.

Method	Sample	Γ (Jm^{-2})	Ref
Intercalation of nanoparticles	FL	0.482	[39]
Pressurized blister	1L	0.212±0.037	[42]
	2L	0.166±0.004	
	3L	0.237±0.016	
	1L CVD	0.236±0.021	
Wrinkle	FL	0.170±0.033	[43]
Interfacial nanoblisters	1L	0.082±0.001	[41]

Supplementary Note 5.3. Summary of the cohesion energy

In order to provide a valuable guideline for the fabrication of vdW heterostructures based on the vdW pick-up transfer techniques, we present a summary of the cohesion energy at the intact G, hBN and MoS_2 homointerfaces (**Supplementary Table 5**) and the interfacial adhesion energy of untreated and precooling-treated homo/heterostructures (**Supplementary Table 6**), corresponding to the experimental data points in **Fig. 1e** and **Fig. 2** of the main text, respectively.

Supplementary Table 5. Summary of cohesion energy Γ (Jm^{-2}) at intact G, hBN and MoS_2 homointerfaces.

T(°C)	Normal force microscopy technique			Shear force microscopy technique		
	G/G	hBN/hBN	MoS_2/MoS_2	G/G	hBN/hBN	MoS_2/MoS_2
-15	0.336±0.025	0.319±0.022	0.471±0.035			
22	0.328±0.028	0.326±0.026	0.482±0.032	0.361±0.014	0.372±0.015	0.501±0.017
130	0.324±0.027	0.322±0.029	0.479±0.036			
300	0.333±0.026	0.312±0.027	0.484±0.030			

Supplementary Table 6. Interfacial adhesion energy Γ (Jm^{-2}) in similar/dissimilar heterostructures using normal force microscopy.

T(°C)	Similar vdW heterostructures			Dissimilar vdW heterostructures			2D crystal/SiO_x heterostructures		
	G/G	hBN/hBN	MoS_2/MoS_2	G/hBN	MoS_2/hBN	MoS_2/G	G/SiO_x	hBN/SiO_x	MoS_2/SiO_x
	**Untreated substrates**								
-15	0.239±0.044	0.250±0.035	0.391±0.045	0.251±0.031	0.279±0.033	0.266±0.034	0.223±0.052	0.200±0.043	0.174±0.034
22	0.230±0.035	0.259±0.032	0.384±0.042	0.248±0.035	0.262±0.025	0.269±0.038	0.239±0.054	0.208±0.047	0.189±0.027
130	0.245±0.034	0.265±0.031	0.401±0.037	0.265±0.033	0.290±0.030	0.290±0.027	0.259±0.045	0.228±0.031	0.205±0.043
300	0.268±0.028	0.296±0.044	0.417±0.050	0.304±0.029	0.288±0.047	0.281±0.048	0.276±0.044	0.235±0.041	0.211±0.038
	**Precooling-treated substrates**								
-15	0.265±0.030	0.276±0.027	0.416±0.036	0.271±0.024	0.270±0.027	0.283±0.025	0.171±0.041	0.177±0.042	0.152±0.034
22	0.270±0.031	0.282±0.026	0.411±0.037	0.264±0.025	0.278±0.023	0.265±0.028	0.209±0.044	0.186±0.043	0.169±0.039
130	0.303±0.028	0.297±0.038	0.445±0.036	0.295±0.034	0.327±0.029	0.321±0.026	0.273±0.043	0.220±0.038	0.209±0.046
300	0.313±0.026	0.310±0.042	0.455±0.038	0.312±0.040	0.331±0.044	0.317±0.037	0.284±0.047	0.230±0.042	0.197±0.048

Supplementary Note 6. Interaction of G with airborne contaminants using water contact angle measurements

In this section, we quantify the effect of surface contaminations and thermal annealing on the IAE of the G crystal by characterizing intrinsic water wettability of fresh and aged surfaces of the G crystal. To this end, the total surface energy of G crystals, γ_G, which consists of contributions from both nonpolar (dispersion) interaction γ_G^d and polar (electrostatic) interaction γ_G^p of graphite (i.e., $\gamma_G = \gamma_G^d + \gamma_G^p$), can be given by the Fowkes equation:

$$2\left(\sqrt{\gamma_W^d \gamma_G^d} + \sqrt{\gamma_W^p \gamma_G^p}\right) = \gamma_W(1 + \cos\theta) \tag{18}$$

where γ_W^d and γ_W^p are the dispersion (21.8 mJ m^{-2}) and polar (51.0 mJ m^{-2}) components of the total surface energy of water γ_W (72.8 mJ m^{-2}), and θ is the WCA of graphite. To determine the electrostatic surface energy of graphite, we first measured the surface electrostatic potential of graphite using a conductive AFM tip (SCM-PIT, Nanoworld, with the spring constant of 1.9 Nm^{-1}) and found it to be ~398 mV for the tip-substrate distance of 10 nm. We then calculated the electrostatic surface energy of graphite γ_G^p (0.07 mJ m^{-2}) using the well-established concept of the parallel plate capacitor model. The interfacial adhesion energy of the G crystal ($\Gamma_G = 2\gamma_G$) in **Fig. 5** in the main text and **Supplementary Table 7** is calculated by finding the dispersion interaction as follows

$$\gamma_G^d = \frac{1}{\gamma_W^d}\left[\frac{\gamma_W}{2}(1 + \cos\theta) - \sqrt{\gamma_W^p \gamma_G^p}\right]^2 \tag{19}$$

The results marked by the star in **Supplementary Table 7** are the IAE of airborne contamination adlayer, γ_{CHO} ($= \gamma_{CHO}^d + \gamma_{CHO}^p$), whose electrostatic interaction γ_{CHO}^p was similarly determined to be 0.32 mJ m^{-2} and therefore can be expressed by

$$\gamma_{CHO}^d = \frac{1}{\gamma_W^d}\left[\frac{\gamma_W}{2}(1 + \cos\theta) - \sqrt{\gamma_W^p \gamma_{CHO}^p}\right]^2 \tag{20}$$

Supplementary Table 7. Summary of water contact angle measurements and corresponding IAE values of G crystal.

Notes	Measured within	WCA	$2\gamma_G$ (Jm^{-2})	Ref
Ultrahigh vacuum	3 sec	42±7°	0.348±0.033	[44]
Ambient air at 24 °C/48% RH	10 sec	64.4°	0.232	[45]
	2 days	91.0°	0.093*	
550°C annealing in Ar	1 min	54.1°	0.286	
Ambient air at 22-25 °C/20-40% RH	10 sec	64.4±2.9°	0.232±0.015	[46]
	7 days	97.0±1.8°	0.072±0.01*	
Ultrahigh vacuum for 15 h	N.A.	59°	0.260	
Ambient air at 22-25 °C/20-40% RH	10 sec	68.6±7.1°	0.210±0.034	[47]
Ambient air at 22 °C/50% RH	10 sec	68.2±2°	0.212±0.010	[48]

	1 day	90±0.1°	0.096±0.002*	
Ambient air at RT/40-50% RH	1.5 min	62.9±2.2°	0.239±0.012	[49]
Ambient air at RT/40-50% RH	5 min	61.8±3.3°	0.249±0.017	
	1 day	81.9±2.9°	0.129±0.012*	
600 °C annealing in He	N.A.	51.4±2.0°	0.300±0.010	
Clean room at 21 °C/40% RH	5 sec	53±5°	0.292±0.027	[50]
	8 min	66±3°	0.223±0.016	
	2 days	86±4°	0.112±0.016*	
Water vapor atmosphere	N.A.	58±2°	0.266±0.010	
Ultrahigh-purity argon atmosphere	1 min	45±3°	0.333±0.016	
Evacuation/1050 °C annealing/vacuum	1 min	55±1°	0.281±0.005	
Evacuation/1000 °C annealing/atmosphere	1 min	73±5°	0.187±0.025	
Ambient air at 22-25 °C/20-40% RH	10 sec	60±0.1°	0.255±0.002	[51]

Supplementary Note 7. Calculations of bending stiffness in 2D crystals

A direct measurement of in-plane elastic modulus of monolayer G (342±8 Nm^{-1} [16]), bilayer G (645±16 Nm^{-1} [16]), monolayer hBN (289±24 Nm^{-1} [16]) and monolayer MoS_2 (180±60 Nm^{-1} [17], 120±30 Nm^{-1} [18]) was reported by AFM nanoindentation of suspended 2D crystal membranes. Also, the bending stiffness of monolayer G (1.49 eV) [19], monolayer hBN (1.34 eV) [19] and monolayer MoS_2 (11.7 eV) [18] is obtained by first principles calculations, whose in-plane elastic modulus of monolayer 2D crystals is consistent with the aforementioned experimental values. In addition, the bending stiffness of bilayer G (35.5 eV) was calculated by measuring the critical voltage for snap-through of pre-buckled graphene membranes [20]. This value for the bilayer G also lies within the range of 3.15-110.4 eV predicted by a modified classical plate theory for the effective bending rigidity of multilayer graphene and 2D materials [53].

Supplementary References

[1] H. Rokni and W. Lu, "Nanoscale probing of interaction in atomically thin layered materials," ACS Central Science, pp. 288-297, 2018.

[2] A. Çolak, H. Wormeester, H. J. W. Zandvliet and B. Poelsema, "Surface adhesion and its dependence on surface roughness and humidity measured with a flat tip," Applied Surface Science, vol. 258, no. 18, pp. 6938-6942, 2012.

[3] G. A. Matei, E. J. Thoreson, J. R. Pratt and et al., "Precision and accuracy of thermal calibration of atomic force microscopy cantilevers," Review of Scientific Instruments, vol. 77, p. 083703, 2006.

[4] J. Hutter, "Comment on Tilt of Atomic Force Microscope Cantilevers: Effect on Spring Constant and Adhesion Measurements," Langmuir, vol. 21, p. 2630, 2005.

[5] R. Proksch, T. E. Schäffer, J. P. Cleveland, R. C. Callahan and M. B. Viani, "Finite optical spot size and position corrections in thermal spring constant calibration," Nanotechnology, vol. 15, pp. 1344-1350, 2004.

[6] J. E. Sader, J. W. M. Chon and P. Mulvaney, "Calibration of rectangular atomic force microscope cantilevers," Review of Scientific Instruments, vol. 70, p. 3967, 1999.

[7] W. Wang, S. Dai, X. Li, J. Yang, D. J. Srolovitz and Q. Zheng, "Measurement of the cleavage energy of graphite," Nature Communications, vol. 6, p. 7853, 2015.

[8] B. Li, J. Yin, X. Liu, H. Wu, J. Li, X. Li and W. Guo, "Probing van der Waals interactions at two-dimensional heterointerfaces," Nature Nanotechnology, vol. 10.10138, pp. s41565-019-0405-2, 2019.

[9] D. Li, S. Wi, M. Chen, B. Ryu and X. Liang, "Nanoimprint-assisted shear exfoliation plus transfer printing for producing transition metal dichalcogenide heterostructures," Journal of Vacuum Science & Technology B, vol. 34, p. 06KA01, 2016.

[10] M. Chen, H. Nam, H. Rokni, S. Wi, J. S. Yoon, P. Chen, K. Kurabayashi, W. Lu and X. Liang, "Nanoimprint-assisted shear exfoliation (NASE) for producing multilayer MoS2 structures as field-effect transistor channel arrays," ACS Nano, vol. 9, no. 9, pp. 8773-8785, 2015.

[11] W. G. Cullen, M. Yamamoto, K. M. Burson, J. H. Chen, C. Jang, L. Li, M. S. Fuhrer and E. D. Williams, "High-Fidelity Conformation of Graphene to SiO2 Topographic Features," Physical Review Letters , vol. 105, p. 215504, 2010.

[12] C. H. Lui, L. Liu, K. F. Mak, G. W. Flynn and T. F. Heinz, "Ultraflat graphene," Nature, vol. 462, p. 339–341, 2009.

[13] W. Wang, S. Dai, X. Li, J. Yang, D. J. Srolovitz and Q. Zheng, "Measurement of the cleavage energy of graphite," Nature Communications, vol. 6, p. 7853, 2015.

[14] C. C. Vu, S. Zhang, M. Urbakh, Q. Li, Q. C. He and Q. Zheng, "Observation of normal-force-independent superlubricity in mesoscopic graphite contacts," Physical Review B, vol. 94, p. 081405, 2016.

[15] E. Koren, E. Lörtscher, C. Rawlings, A. W. Knoll and U. Duerig, "Adhesion and friction in mesoscopic graphite contacts," Science, vol. 348, no. 6235, pp. 679-683, 2015.

[16] A. Falin, Q. Cai, E. J. G. Santos, D. Scullion, D. Qian, R. Zhang, Z. Yang, K. Huang and et al., "Mechanical properties of atomically thin boron nitride and the role of interlayer interactions," *Nature Communications,* vol. 8, p. 15815, 2017.

[17] S. Bertolazzi, J. Brivio and A. Kis, "Stretching and Breaking of Ultrathin MoS_2," *ACS Nano,* vol. 5, no. 12, p. 9703–9709, 2011.

[18] R. C. Cooper, C. Lee, C. A. Marianetti, X. Wei, J. Hone and J. W. Kysar, "Nonlinear elastic behavior of two-dimensional molybdenum disulfide," *Physical Review B,* vol. 87, p. 035423, 2013.

[19] K. N. Kudin and G. E. Scuseria, "C_2F, BN, and C nanoshell elasticity from ab initio computations," *Physical Review B,* vol. 64, p. 235406, 2001.

[20] N. Lindah, D. Midtvedt, J. Svensson, O. A. Nerushev, N. Lindvall, A. Isacsson and E. E. B. Campbell, "Determination of the Bending Rigidity of Graphene via Electrostatic Actuation of Buckled Membranes," *Nano Letters,* vol. 12, no. 7, p. 3526–3531, 2012.

[21] L. A. Girifalco and R. A. Lad, "Energy of cohesion, compressibility, and the potential energy functions of the graphite system," Journal of Chemical Physics, vol. 25, pp. 693-697, 1956.

[22] L. X. Benedict, N. G. Chopra, M. L. Cohen, A. Zettl, S. G. Louie and V. H. Crespi, "Microscopic determination of the interlayer binding energy in graphite," Chemical Physics Letters, vol. 286, no. 5-6, pp. 490-496, 1998.

[23] R. Zacharia, H. Ulbricht and T. Hertel, "Interlayer cohesive energy of graphite from thermal desorption of polyaromatic hydrocarbons," Physical Review B, vol. 69, p. 155406, 2004.

[24] A. Kis, K. Jensen, S. Aloni, W. Mickelson and A. Zettl, "Interlayer Forces and Ultralow Sliding Friction in Multiwalled Carbon Nanotubes," Physical Review Letters, vol. 97, no. 2, p. 025501, 2006.

[25] Z. Liu, J. Z. Liu, Y. Cheng, Z. Li, L. Wang and Q. Zheng, "Interlayer binding energy of graphite: A mesoscopic determination from deformation," Physical Review B, vol. 85, p. 205418, 2012.

[26] Z. Deng, A. Smolyanitsky, Q. Li, X. Q. Feng and R. J. Cannara, "Adhesion-dependent negative friction coefficient on chemically-modified graphite at the nanoscale," Nature Materials, vol. 14, pp. 714-720, 2012.

[27] R. Zhang, Z. Ning, Y. Zhang, Q. Zheng, Q. Chen, H. Xie, Q. Zhang, W. Qian and F. Wei, "Superlubricity in centimeters-long double-walled carbon nanotubes under ambient conditions," Nature Nanotechnology, vol. 8, pp. 912-916, 2013.

[28] M. R. Roenbeck, X. Wei, A. M. Beese, M. Naraghi, A. Furmanchuk, J. T. Paci, G. C. Schatz and H. D. Espinosa, "In Situ Scanning Electron Microscope Peeling To Quantify Surface Energy between Multiwalled Carbon Nanotubes and Graphene," Acs Nano, vol. 8, no. 1, pp. 124-138, 2014.

[29] P. Li, Z. You and T. Cui, "Adhesion energy of few layer graphene characterized by atomic force microscope," Sensors and Actuators A: Physical, vol. 217, pp. 56-61, 2014.

[30] J. Wang, D. C. Sorescu, S. Jeon, A. Belianinov, S. V. Kalinin, A. P. Baddorf and P. Maksymovych, "Atomic intercalation to measure adhesion of graphene on graphite," Nature Communications, vol. 7, p. 13263, 2016.

[31] C. D. V. Engers, N. E. A. Cousens, V. Babenko, J. Britton, B. Zappone, N. Grobert and S. Perkin, "Direct measurement of the surface energy of graphene," Nano Letters, vol. 17, pp. 3815-3821, 2017.

[32] S. P. Koenig, N. G. Boddeti, M. L. Dunn and J. S. Bunch, "Ultrastrong adhesion of graphene membranes," Nature Nanotechnology, vol. 6, pp. 543-546, 2011.

[33] i. N. G. Boddet, S. P. Koenig, R. Long, J. L. Xiao, J. S. Bunch and M. L. Dunn, "Mechanics of adhered, pressurized graphene blisters," Journal of Applied Mechanics, vol. 80, p. 040909, 2013.

[34] N. G. Boddeti, X. Liu, R. Long, J. L. Xiao, J. S. Bunch and M. L. Dunn, "Graphene blisters with switchable shapes controlled by pressure and adhesion," Nano Letters, vol. 13, no. 12, pp. 6216-6221, 2013.

[35] T. Jiang and Y. Zhu, "Measuring graphene adhesion using atomic force microscopy with a microsphere tip," Nanoscale, vol. 7, p. 10760, 2015.

[36] Z. Zhong, C. L. Chen, M. R. Dokmeci and K. T. Wan, "Direct measurement of graphene adhesion on silicon surface by intercalation of nanoparticles," Journal of Applied Physics, vol. 107, p. 026104, 2010.

[37] S. R. Na, J. W. Suk, R. S. Ruoff, R. Huang and K. M. Liechti, "Ultra long-range interactions between large area graphene and silicon," ACS Nano, vol. 8, no. 11, pp. 11234-11242, 2014.

[38] X. Gao, X. Yu, B. Li, S. Fan and C. Li, "Measuring graphene adhesion on silicon substrate by single and dual nanoparticle-loaded blister," Advanced Materials Interfaces, vol. 4, no. 9, p. 1601023, 2017.

[39] J. Torres, Y. Zhu, P. Liu, S. C. Lim and M. Yun, "Adhesion energies of 2D graphene and MoS2 to silicon and metal substrates," Physica Status Solidi A, vol. 215, no. 1, p. 1700512, 2018.

[40] S. J. Cartamil-Bueno, A. Centeno, A. Zurutuza, P. G. Steeneken, H. S. J. van der Zanta and S. Houri, "Very large scale characterization of graphene mechanical devices using a colorimetry technique," Nanoscale, vol. 9, p. 7559, 2017.

[41] D. A. Sanchez, Z. Dai, P. Wang, A. Cantu-Chavez, C. J. Brennan, R. Huang and N. Lu, "Mechanics of spontaneously formed nanoblisters trapped by transferred 2D crystals," PNAS, vol. 115, no. 31, pp. 7884-7889, 2018.

[42] D. Lloyd, X. Liu, N. Boddeti, L. Cantley, R. Long, M. L. Dunn and J. S. Bunch, "Adhesion, Stiffness, and Instability in Atomically Thin MoS2 Bubbles," Nano Letters, vol. 17, no. 9, pp. 5329-5334, 2017.

[43] S. Deng, E. Gao, Z. Xu and V. Berry, "Adhesion energy of MoS2 thin films on silicon-based substrates determined via the attributes of a single wrinkle," ACS Applied Materials Interfaces, vol. 9, no. 8, pp. 7812-7818, 2017.

[44] M. E. Schrader, "Ultrahigh-Vacuum Techniques in the Measurement of Contact Angles. 5. LEED Study of the Effect of Structure on the Wettability of Graphite," Journal of Physical Chemistry, vol. 84, p. 2774−2779, 1980.

[45] Z. Li, Y. Wang, A. Kozbial, G. Shenoy, F. Zhou, R. McGinley, P. Ireland, B. Morganstein, A. Kunkel, S. P. Surwade, L. Li and H. Liu, "Effect of Airborne Contaminants on the Wettability of Supported Graphene and Graphite," Nature Materials, vol. 12, pp. 925-931, 2013.

[46] A. Kozbial, Z. Li, J. Sun, X. Gong, F. Zhou, Y. Wang, H. Xu, H. Liu and L. Li, "Understanding the Intrinsic Water Wettability of Graphite," Carbon, vol. 74, p. 218−225, 2014.

[47] A. Kozbial, C. Trouba, H. Liu and L. Li, "Characterization of the Intrinsic Water Wettability of Graphite Using Contact Angle Measurements: Effect of Defects on Static and Dynamic Contact Angles," Langmuir, vol. 33, p. 959−967, 2017.

[48] C. A. Amadei, C. Y. Lai, D. Heskes and M. Chiesa, "Time Dependent Wettability of Graphite Upon Ambient Exposure: The Role of Water Adsorption," Journal of Chemical Physics, vol. 141, p. 084709, 2014.

[49] Y. Wei and C. Q. Jia, "Intrinsic Wettability of Graphitic Carbon," Carbon , vol. 87, p. 10−17, 2015.

[50] A. Ashraf, Y. Wu, M. C. Wang, N. R. Aluru, S. A. Dastgheib and S. Nam, "Spectroscopic Investigation of the Wettability of Multilayer Graphene Using Highly Ordered Pyrolytic Graphite as a Model Material," Langmuir, vol. 30, p. 12827−12836, 2014.

[51] Z. Li, A. Kozbial, N. Nioradze, D. Parobek, G. J. Shenoy, M. Salim, S. Amemiya, L. Li and H. Liu, "Water Protects Graphitic Surface from Airborne Hydrocarbon Contamination," ACS Nano, vol. 10, no. 1, p. 349–359, 2016.

[52] Y. Gongyang, C. Qu, S. Zhang, M. Ma and Q. Zheng, "Eliminating delamination of graphite sliding on diamond-like carbon," Carbon, vol. 132, p. 444−450, 2018.

[53] G. Wang, Z. Dai, J. Xiao, S. Feng, C. Weng, L. Liu, Z. Xu, R. Huang and Z. Zhang, "Bending of Multilayer van der Waals Materials," Physical Review Letters, vol. 123, 116101, 2019.
